# Predicting recurrent atrial fibrillation after catheter ablation: a systematic review of prognostic models

**DOI:** 10.1093/europace/euaa041

**Published:** 2020-03-30

**Authors:** Janine Dretzke, Naomi Chuchu, Ridhi Agarwal, Clare Herd, Winnie Chua, Larissa Fabritz, Susan Bayliss, Dipak Kotecha, Jonathan J Deeks, Paulus Kirchhof, Yemisi Takwoingi

**Affiliations:** e1 Institute of Applied Health Research, University of Birmingham, Birmingham B15 2TT, UK; e2 Institute of Cardiovascular Sciences, University of Birmingham, Birmingham B15 2TT, UK; e3 University Hospitals Birmingham NHS Foundation Trust, Birmingham B15 2GW, UK; e4 Sandwell and West Birmingham Hospitals NHS Trust, Birmingham B18 7QH, UK

**Keywords:** Atrial fibrillation, Catheter ablation, Recurrence, Prognostic model, Model performance, Systematic review

## Abstract

**Aims:**

We assessed the performance of modelsf (risk scores) for predicting recurrence of atrial fibrillation (AF) in patients who have undergone catheter ablation.

**Methods and results:**

Systematic searches of bibliographic databases were conducted (November 2018). Studies were eligible for inclusion if they reported the development, validation, or impact assessment of a model for predicting AF recurrence after ablation. Model performance (discrimination and calibration) measures were extracted. The Prediction Study Risk of Bias Assessment Tool (PROBAST) was used to assess risk of bias. Meta-analysis was not feasible due to clinical and methodological differences between studies, but c-statistics were presented in forest plots. Thirty-three studies developing or validating 13 models were included; eight studies compared two or more models. Common model variables were left atrial parameters, type of AF, and age. Model discriminatory ability was highly variable and no model had consistently poor or good performance. Most studies did not assess model calibration. The main risk of bias concern was the lack of internal validation which may have resulted in overly optimistic and/or biased model performance estimates. No model impact studies were identified.

**Conclusion:**

Our systematic review suggests that clinical risk prediction of AF after ablation has potential, but there remains a need for robust evaluation of risk factors and development of risk scores.

**What’s new?**

- Several prognostic models have been developed to predict individual risk of recurrence of atrial fibrillation (AF) after catheter ablation. To the best of our knowledge this is the first comprehensive systematic review of such models to (i) include detailed risk of bias assessment of model development and validation studies and (ii) provide a descriptive summary of measures of model performance in forest plots.
- Model discriminatory ability based on the c-statistic was highly variable; no model had consistently poor or good discriminatory ability. Model calibration (i.e. how well predicted risk agrees with observed risk) was rarely reported. Thus overall assessment of model performance remains incomplete.
- Risks of bias were substantial and included a lack of internal validation in model development studies, flawed variable selection and weighting, low event rates and poor reporting of missing data.
- Robust evaluation of risk factors and development of clinically useful risk scores is still needed.

## Introduction

Atrial fibrillation (AF) is the most common arrhythmia diagnosed in clinical practice, and worldwide incidence and prevalence is increasing.^1^ Atrial fibrillation is predicted to affect between 1.3 and 1.8 million patients in the UK and 18 million people in Europe by 2060.^2^^,^^3^ Drivers for this increase include an ageing population, better survival from conditions such as ischaemic heart disease and increasing multimorbidity.^4^^,^^5^ Atrial fibrillation is associated with increased morbidity and mortality, particularly cardiovascular related.^4^^,^^5^ Currently available treatments can reduce this, particularly via anticoagulation for stroke prevention,^5^ but many patients remain symptomatic even on optimal rate control therapy. Furthermore, these patients remain at high risk of cardiovascular complications, often manifesting as heart failure or sudden death.^6^^,^^7^ To mitigate this epidemic of AF-related disease, efforts are underway to improve primary and secondary prevention.^8^^,^^9^

Unfortunately, recurrent AF is common: approximately 70% of patients experience recurrence after a cardioversion.^10^^,^^11^ This proportion can be somewhat reduced with the use of antiarrhythmic drugs.^10^^,^^11^ Atrial fibrillation ablation, mainly via pulmonary vein isolation, is an effective and safe intervention to restore and maintain sinus rhythm.^12^^,^^13^ Recurrence of AF after catheter ablation is estimated to be between 20% and 45%.^14^^,^^15^ Catheter ablation seems to achieve a better quality of life than antiarrhythmic drug therapy.^16^^,^^17^ Furthermore, recent data suggest that AF ablation could have a positive effect on left ventricular function in patients with heart failure.^18^ These benefits are better sustained in patients who remain free of AF and need to be balanced against the discomfort and complication risk of AF ablation.^5^ Hence, there is a growing clinical need to identify patients at risk of developing recurrent AF after AF ablation.

Numerous risk factors are associated with the development of AF, including age, hypertension, diabetes mellitus, and heart failure.^19^^,^^20^ Less validated risk factors include subclinical hyperthyroidism, obesity, and sleep apnoea syndrome.^19^ Risk factors associated with recurrence are less well-established but likely include type of AF (chronic or paroxysmal) and echocardiographic parameters.^21^^,^^22^

Prognostic models, which combine several predictors to generate an individualized risk estimate have been developed for AF prediction in different populations. We identified two systematic reviews on prognostic models for predicting recurrent AF after ablation^23^^,^^24^; these reviews had limited search strategies and did not include formal risk of bias appraisal. We therefore performed a comprehensive systematic review on predicting recurrent AF in patients who underwent AF ablation.

## Methods

The systematic review protocol was registered with PROSPERO (CRD42018111649). Full details of methods have been published.^25^

### Study eligibility criteria

#### Study design

Published or unpublished studies reporting (i) prediction model development with internal validation, (ii) prediction model development with external validation, (iii) external model validation with or without model updating, or (iv) model impact assessment were eligible for inclusion. Studies that developed a new model with no subsequent validation were recorded but not assessed. A prognostic model was defined as a combination of two or more predictors within a statistical model used to predict an individual’s risk of the outcome.^26^ An impact study quantifies the impact of the model on clinical decision-making and patient outcome.

#### Population

Patients undergoing single or repeat ablation using any method were eligible for inclusion. There were no restrictions on previous treatments.

#### Outcomes

The clinical outcome of interest was recurrent AF at any time post-ablation. We excluded models that were developed for predicting a different outcome (e.g. the CHADS_2_ score for stroke prediction). Model performance measures of interest were calibration measures (e.g. calibration slope, calibration-in-the-large), which indicate how well the predicted risk compares to the observed risk, and discrimination measures (e.g. c-statistic), which indicate how well the model differentiates between those with and without the outcome.^27^ Measures that quantify the added discriminative value of one model over another, such as the net reclassification index (NRI) and/or integrated discrimination index (IDI), were also extracted.

### Search strategy

Bibliographic databases (MEDLINE, MEDLINE In-Process, Embase, and Cochrane CENTRAL) were searched from inception to November 2018 using combinations of text and index terms relating to AF and models (Supplementary material online, *File S1*). The ‘model’ component of the search strategy was informed by a validated search filter.^28^ There were no date or language restrictions. Reference lists of relevant articles were checked and subject experts consulted. ClinicalTrials.gov and the WHO International Clinical Trials Registry Platform were searched for ongoing studies and the Conference Proceedings Citation Index for conference abstracts.

### Study selection

A sample of records was screened by two reviewers to pilot the screening criteria. In a change from the protocol, the remainder of the title and abstract screening was undertaken by one reviewer only (J.D., N.C., or C.H.) to process the large volume of records retrieved (*n* = 16 023). Records, where eligibility for inclusion was unclear, were discussed by a panel of reviewers (J.D., N.C., Y.T., and C.H.), and disagreements on study eligibility were resolved through discussion. Full texts (*n* = 150) were reviewed where a decision could not be made based on title and abstract.

### Data extraction

Data extraction was undertaken by one reviewer (J..D.) using a pre-defined and piloted data extraction form (Excel 2016). Data items to extract were based on the CHARMS^29^ checklist, and included:

- Participants (e.g. proportion with paroxysmal/persistent AF, ablation procedure).
- Study design (e.g. prospective or retrospective cohort, sample size, length of follow-up).
- Outcome measures (e.g. definition and frequency of outcome assessment).
- Model development (e.g. method for selection of predictors, validation method).
- Model performance (e.g. c-statistic, ratio of observed and expected events (*E/O*)).

### Assessment of risk of bias

Risk of bias was assessed using the Prediction Study Risk Of Bias Assessment Tool (PROBAST).^30^ This assesses criteria within five domains: participant selection; predictors; outcomes; sample size; and patient flow and analysis (Supplementary material online, *File S2*). Risk of bias assessment was performed by one reviewer (J.D.) and checked by a further two (Y.T. and R.A.).

### Synthesis

All studies were narratively described, with key findings tabulated and results presented with confidence intervals (CIs) when reported. Several studies reported the c-statistic. However, quantitative pooling was not possible due to differences in populations (e.g. different approaches to ablation, single vs. repeat ablation), variable electrocardiogram (ECG) monitoring intensity for recurrent AF,^31^ length of follow-up, possible overlap between patient cohorts, and a lack of uncertainty measures such as CIs. The c-statistics, grouped by type of model or by study, were instead presented in forest plots; this included subgroup analyses. A c-statistic of ≥0.7 was considered good and ≥0.8 very good discriminative ability; values <0.7 were considered weak, and <0.5 as very weak.^32^ These cut-offs are arbitrary and intended as a rough guide only. Lack of meta-analysis precluded formal exploration of publication bias using funnel plots.

The body of evidence identified was considered in the context of the Grading of Recommendations, Assessment, Development and Evaluations (GRADE)^33^ domains (risk of bias, imprecision, inconsistency, indirectness, and publication bias). As there is no specific guidance on how to apply GRADE to systematic reviews of prognostic models, we did not produce a GRADE summary of findings table or generate a quality score. PRISMA guidelines^34^ were followed for the reporting of the systematic review.

## Results

### Search results

**Figure 1:**
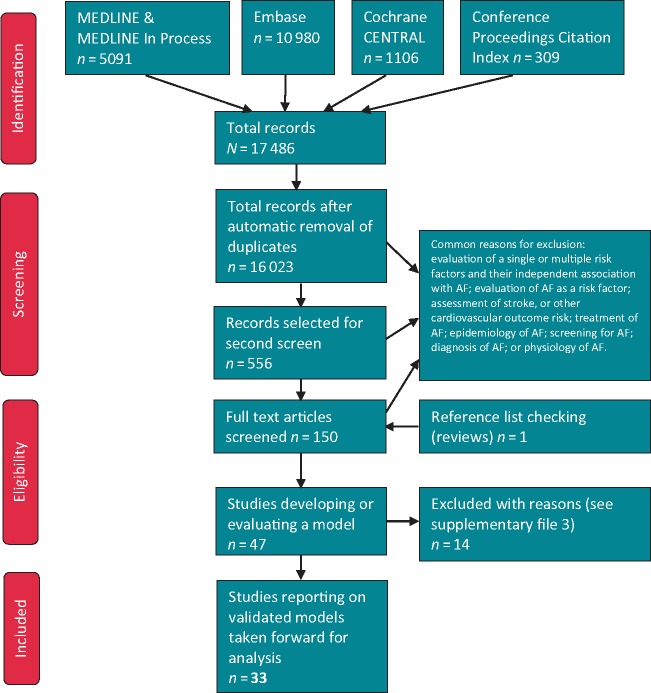
PRISMA flow diagram. AF, atrial fibrillation.

Thirty-three studies of 13 models were included (*Figure 1*). Six studies^35–40^ included two separate cohorts. Studies that developed a model, which was not validated (either in the same or another study) were documented but not analysed (Supplementary material online, *File S3*). One study (Kosiuk *et al*.^41^) developed and externally validated a score (DR-FLASH) primarily to predict low-voltage areas rather than AF; this study was not included but findings have been presented in Supplementary material online, *File S4*.

### Study characteristics

Twelve studies^37–40^^,^^42–49^ described the development (or modification) of a model, and 28 studies^35–40^^,^^44^^,^^46^^,^^47^^,^^50–68^ (including 31 patient cohorts) undertook external model validation. Seven^37–40^^,^^44^^,^^46^^,^^47^ of these studies undertook both model development and external validation. Twenty-five studies reported a single relevant model, and eight studies^36^^,^^37^^,^^44^^,^^46^^,^^55^^,^^64^^,^^66^^,^^67^ evaluated two or more. No model impact studies were identified. Most studies were retrospective analyses of consecutive patients; detailed study characteristics are provided in Supplementary material online, *File S5*.

### 

#### Variables included in models

Twenty-five variables were included across 13 models (*Table 1*). Models included between three and six variables. The most common variables were left atrial parameters (nine models), type of AF (eight models), age (seven models), sex (four models), and estimated glomerular filtration rate (eGFR, four models).

**Table 1:** Model variables

Variables	Risk score Berkowitsch 2012	ALARMEc	APPLE	SUCCESS	ATLAS	BASE-AF_2_	CAAP-AF	HATCH	HATCH + OSA	B-HATCH	MB-LATER	FER2CI	Risk score Jarman 2012
Age			√	√	√		√	√	√	√			
Sex					√		√				√	√	
Type of AF	√	√	√	√	√	√	√				√		
Duration of persistent AF													√
Previous ablations				√									
MetS	√	√											
eGFR	√	√	√	√									
Left atrial parameters	√	√	√	√	√	√	√				√		√
Min coupling interval of APC												√	
Cardiomyopathy		√											
Heart failure								√	√	√			
LVEF			√	√									
BMI						√							
Current smoking					√	√							
AF history						√							
Early recurrence						√					√	√	
CAD							√						
Antiarrhythmics failed							√						
Hypertension								√	√	√			
OSA									√				
COPD								√	√	√			
Stroke or TIA								√	√	√			
BNP										√			
Bundle branch block											√		
Presence of severe comorbidity^a^													√
Point range and cut-offs;1 point for each variable unless otherwise stated	Point range: 0–4; √ non-PAF; √ ≤68 mL/min eGFR; √ NLA >11.5	Point range: 0–5; √ non-PAF; √ ≤68 mL/min eGFR; √ NLA >11.5 or >10.25 depending on study	Point range: 0–5; √ >65 years; √ PAF; √ <60 mL/min eGFR; √ LAD ≥43 mm; √ <50% LVEF	Point range as APPLE score + additional point for each previous ablation	Possible points: 15+; 1 for age (>60); 4 for female gender; 7 for current smoker; 2 for non-PAF; 1 for each 10 mL/m^2^ LAV indexed for body surface area	Point range: 0–6; √ non-PAF; √ LAD >40 mm; √ >28 kg/m^2^ BMI; √ >6 years AF	Point range: 0–13; 1 for <50, 2 for 50 to <60, 3 for 60 to <70, and 4 for ≥70; √ female; 2 for persistent or long-standing AF; 0 for LAD <4; 1 for 4 to <4.5; 2 for 4.5 to <5; 3 for 5 to <5.5; and 4 for ≥5.5 cm; 0 for none, 1 for 1 or 2, 2 for >2 (AADs failed)	Point range: 0–7; √ >75 years; 2 for stroke and heart failure	No details on point range; √ >75 years; 2 for stroke and heart failure	Point range: 0–10; √ >75 years; 2 for stroke and heart failure; 3 points for BNP ≥100 pg/dL	Point range: 0–6; 1 for PAF and 2 for long-standing AF; √ male; √ LAD ≥47 mm	Point range: 0–4; 1 point for female; 2 for early recurrence of AF; 1 for coupling interval <49%	Point range: 0–7; √ duration of continuous AF >1 year (1 point); √ LAD 40–45 mm (1 point), 46–50 mm (2 points), >50 (3 points); √ any severe comorbidity^a^ 3 points

√, variable included in model; AADs, antiarrhythmic drugs; AF, atrial fibrillation; APC, atrial premature contraction; BMI, body mass index; BNP, brain natriuretic peptide; CAD, coronary artery disease; COPD, chronic obstructive pulmonary disease; eGFR, estimated glomerular filtration rate; LAD, left atrial diameter; LVEF, left ventricular ejection fraction; MetS, metabolic syndrome; NLA, normalized left atrial area; OSA, obstructive sleep apnoea; PAF, paroxysmal atrial fibrillation; TIA, transient ischaemic attack.

a Severe comorbidity defined as severe mitral regurgitation, moderate mitral stenosis, mitral valvotomy, mitral valve replacement, hypertrophic cardiomyopathy, or structural congenital heart disease.

### Risk of bias

#### Population, predictors, and outcomes

**Figure 2:**
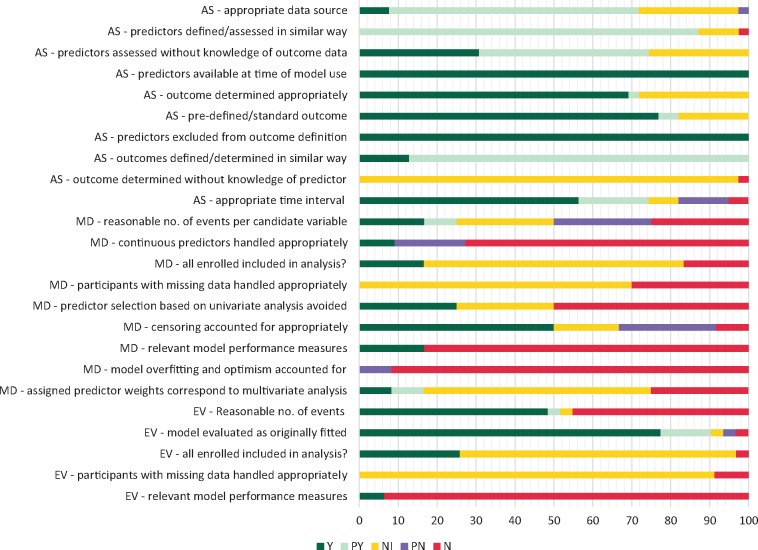
Risk of bias summary. Chart shows percentage of study cohorts meeting/not meeting criteria: AS, all studies (39 study cohorts); EV, external validation studies (31 study cohorts); MD, model development studies (12 study cohorts); N, no; NI, no or insufficient information; PN, probably no; PY, probably yes; Y, yes. There are more evaluations than studies, as some studies included more than one cohort and/or analysis; the criterion ‘participants with missing data handled appropriately’ is only applicable where there was missing data.

There was poor reporting of whether AF recurrence was determined without knowledge of predictor information (97% of study cohorts; *Figure 2*). Only one study^46^ specifically noted that treating physicians were not blinded to one of the variables [brain natriuretic peptide (BNP) status] which may have influenced frequency or intensity of screening. Studies did not always report how AF recurrence was assessed (28% of study cohorts), whether a standard outcome definition was used (18%) or whether predictors were assessed without knowledge of outcome information (26%). An assumption was made that single-centre studies would have a consistent approach to defining and assessing predictors, although this may not always be the case (e.g. for left atrial parameters). Studies used a combination of ECG and Holter monitoring for assessing recurrence, with around 60% of studies reporting that additional investigations were scheduled if patients reported symptoms. There was variation both within and between studies in intensity of monitoring which can influence outcome detection (e.g. monitoring between two and four times in the first year). Only one study^44^ reported the proportion of patients who received Holter monitoring. Three studies^56^^,^^60^^,^^67^ had a proportion of patients with implantable recorders and one^40^ a proportion of patients with pacemaker data. Follow-up time was variable (6 months to >5 years, Supplementary material online, *File S5*).

#### Analysis—model development studies

Model development was subject to substantial risk of bias and/or poor reporting (*Figure 2*; Supplementary material online, *File S2*). Three studies (25%)^40^^,^^42^^,^^47^ had an adequate (>10) number of events per candidate variable, and three studies^38^^,^^40^^,^^46^ used appropriate methods for selecting predictors (i.e. based on multivariable modelling). One study^46^ stated that a variable cut-off was chosen on the basis of prior research; the remaining studies appeared to dichotomize at least one variable based on study data. Two studies^47^^,^^49^ (17%) appeared to appropriately assign predictor weights based on regression coefficients; the remaining studies (83%) gave no information or used an incorrect method (such as simply assigning one point per variable). Time-to-event analysis (Cox model) was appropriately used in six (50%) studies.^37^^,^^40^^,^^42^^,^^43^^,^^47^^,^^48^ Eleven (92%) studies did not perform internal validation and thus failed to account for model overfitting and optimism in model performance; one study^47^ used a split sample approach which is not thought to be an adequate method. Five studies^43–46^^,^^50^ (42%) modified existing scores, e.g. by adding another variable, or changing a variable cut-off, but did not consider these as new models or perform internal validation. No analyses were performed of the added value of a modified model compared with the previous one.

#### Analysis—model evaluation studies

Twenty-eight studies (31 cohorts) externally validated a previously developed model. Fifteen cohorts (50%) had sample sizes with event rates of 100 or over, whilst 16 had smaller sample sizes and event rates (<100). Most cohorts (90%) appeared to evaluate models using the same variable cut-offs as specified in the model development study. An exception were studies relating to ALARMEc^50^^,^^51^ where variable cut-offs were changed.

#### Analysis—all studies

For most analyses (70%), there was insufficient information on data completeness. Most studies were based on retrospective analyses and eligibility criteria sometimes related to availability of model variable data and/or a minimum follow-up time but this was not always made explicit. Around 60% of analyses presented a measure of model discrimination (c-statistic). Only two studies^44^^,^^47^ additionally considered model calibration. Neither discrimination nor calibration measures were reported in 30% of analyses.

### Model performance

#### ALARMEc

**Figure 3:**
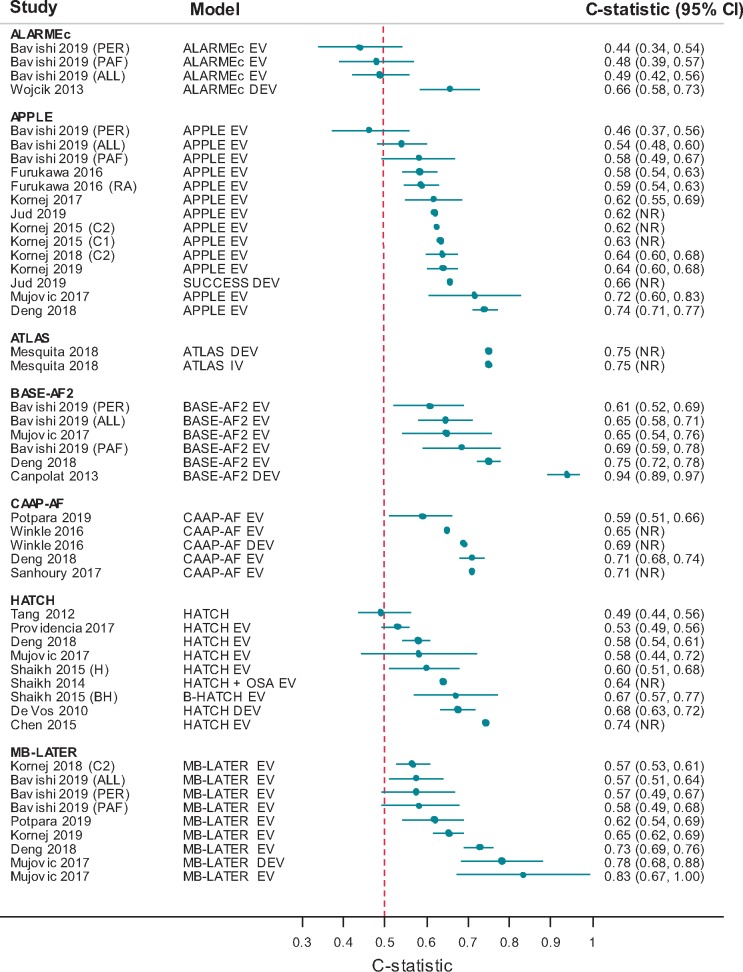
Model c-statistics (by model). ALL, all patients; BH, B-HATCH score; C1, cohort 1; C2, cohort 2; CI, confidence interval; DEV, model development; EV, external model validation; H, HATCH score; PAF, paroxysmal AF sub-group; PER, persistent AF sub-group; RA, repeat ablation sub-group.

Five studies were identified. Berkowitsch *et al*.^42^ developed a risk score [variables: type of AF, metabolic syndrome, eGFR, and normalized left atrial area (NLA)], and applied this to patients undergoing first ablation (Supplementary material online, *File S5*). Subsequent studies added a further variable (cardiomyopathy)^43^ and externally validated the score^43^^,^^50^^,^^51^^,^^67^ in first and/or repeat ablation populations. There was inconsistency in terms of variable cut-off for NLA. Recurrence rates after a first procedure varied between 27% and 47% (Supplementary material online, *File S6*). Four studies found that recurrence increased with increasing risk scores. Two studies reported a c-statistic of 0.66 (95% CI 0.58–0.73)^43^ and 0.49 (95% CI 0.42–0.56),^67^ respectively (*Figure 3*). There was little difference in c-statistic for paroxysmal or persistent AF sub-groups.

#### APPLE

Ten studies^35–37^^,^^44^^,^^52–55^^,^^66^^,^^67^ evaluated the APPLE score [variables: age, type of AF, eGFR, left atrial diameter (LAD), and left ventricular ejection fraction] in first and/or repeat ablation populations (Supplementary material online, *File S5*). A model development study for this risk score was not identified. One study^37^ was specifically interested in very late prediction of recurrence (>12 months). One study (Jud *et al*.^44^) developed a new risk score by adding a variable (previous ablation) to the APPLE score; this new score (SUCCESS) was not internally validated and there was no attempt to quantify the added value of this score compared with APPLE.

Recurrence rates ranged from 16% to 64%. Eight studies reported c-statistics ranging from 0.46 to 0.74 (*Figure 3*) indicating very poor to good discriminative ability. The poorest discriminative ability was in a subgroup of patients with persistent AF.^67^ There was little difference in c-statistic between a repeat ablation subgroup and the total population,^52^ a paroxysmal AF subgroup and total population^67^ or between the APPLE score and the modified APPLE (SUCCESS) score.^44^

One study (Jud *et al*.^44^) reported a calibration measure and found no statistically significant difference between observed and expected events based on the Hosmer–Lemeshow test. This test has limited statistical power and is difficult to interpret as there is no indication of direction or magnitude of miscalibration.^69^ Other measures reported were proportions of recurrence for different scores and odds ratios (Supplementary material online, *File S6*).

#### ATLAS

One study (Mesquita *et al*.^47^) developed and validated this score in patients undergoing first ablation [variables: age, sex, type of AF, current smoking, and indexed left atrial volume]. The recurrence rate was 27%. The c-statistic was 0.75 in both the development and validation cohorts. The calibration-in-the-large-statistic was 0.077 (*P* = 0.272) and the calibration slope 0.93 indicating that observed events were only slightly higher than predicted.

#### BASE-AF_2_

This score was developed by Canpolat *et al*.^48^ and validated in a further three studies.^37^^,^^66^^,^^67^ Included variables were type of AF, LAD, body mass index, current smoking, AF history, and early recurrence. Two studies^37^^,^^67^ had mixed populations in terms of single and repeat ablation. Recurrence rates varied between 15% and 27%. Studies reported c-statistics ranging from 0.61 to 0.94 (*Figure 3*). Sub-group analysis in Bavishi *et al*.^67^ indicated slightly poorer discriminative ability in a persistent AF population [c-statistic 0.61 (95% CI 0.52–0.69)] compared with a paroxysmal AF population [c-statistic 0.69 (95% CI 0.59–0.78)]. A sensitivity of 80% and specificity of 91.6% (CI NR) were reported in the development study^48^ (threshold BASE-AF_2_ ≥ 3).

#### CAAP-AF

The score was developed and externally validated by Winkle *et al*.,^40^ and validated in a further three studies.^56^^,^^64^^,^^66^ Included variables were age, sex, type of AF, LAD, coronary artery disease, and number of antiarrhythmic drugs failed. Most patients were undergoing first ablation.

Recurrence rates varied between 8% and 59%. The studies reported a c-statistic between 0.59 and 0.71 suggesting weak to good discriminative ability (*Figure 3*). Sensitivity and specificity were reported in two studies: 64% and 68% (Sanhoury *et al*.^56^; threshold ≥ 5, CI NR) and 57.9% (95% CI 49.0–66.4) and 57% (95% CI 46.3–67.7) (Potpara *et al*.^64^; threshold ≥ 6), respectively.

#### HATCH

This score was developed for the prediction of progression from paroxysmal to persistent AF in patients who had not undergone ablation (de Vos *et al*.^49^). It has subsequently been applied in 12 studies to predict recurrence of AF in post-ablation cohorts. Included variables are age, heart failure, hypertension, chronic obstructive pulmonary disease, and stroke/transient ischaemic attack. Patients were undergoing first ablation in most studies; three studies^37^^,^^58^^,^^60^ had a proportion with repeat ablation. In two studies,^61^^,^^62^ ablation was performed for atrial flutter rather than AF; however, the model was applied to predict post-ablation AF. One study^37^ used the score to predict very late recurrence (>12 months post-ablation). Shaikh *et al*.^45^ applied a modified version of HATCH (with obstructive sleep apnoea added as variable), and Shaikh *et al*.^46^ evaluated both the HATCH score and a modified version (HATCH + BNP as added variable); neither study performed internal validation of the modified score.

Recurrence rates varied between 16% and 48%. Eight studies reported a c-statistic between 0.49 and 0.74 (*Figure 3*) indicating very poor to good discriminative ability. The remaining studies reported proportion of recurrence according to score and/or mean scores in those with and without recurrence (Supplementary material online, *File S6*). There was no clear trend towards increasing recurrence with higher scores. At a threshold ≥2, the sensitivities and specificities were 25.0% and 92.4% (Miao *et al*.^68^) and 51.8% and 84.7% (Chen *et al*.^61^), respectively.

#### MB-LATER

This score was developed by Mujovic *et al*.^37^ for the prediction of very late recurrence (>12 months) and validated in a very small cohort (*n* = 39). Another five studies^55^^,^^64–67^ applied the score to post-ablation cohorts, with one study^65^ predicting very late recurrence. Included variables are sex, type of AF, LAD, early recurrence, and bundle branch block. Three studies included a proportion of repeat ablations.^37^^,^^64^^,^^67^

Recurrence rates were between 15% and 64%. Five studies reported c-statistics (*Figure 3*) varying between 0.58 and 0.83 indicating very weak to very good discriminative ability. Little difference in c-statistic was reported between paroxysmal [0.58 (95% CI 0.49–0.68)] and persistent AF [0.58 (95% 0.49–0.67)] populations.^67^ Two studies reported sensitivity and specificity of 42.9% (95% CI 34.3–51.7) and 74.2% (95% CI 64.1–82.7) (Potpara *et al*.,^64^ threshold ≥ 2) and 75% and 72.6% (Mujovic *et al*.,^37^ threshold ≥ 2). No calibration measures were reported.

#### Other models

Two additional studies were identified that developed and externally validated a model in separate cohorts, the FER2CI score^39^ [variables: sex, coupling interval of atrial premature contraction, and early recurrence] and a ‘risk score’^38^ [variables: duration of persistent AF, eGFR, and presence of severe comorbidity]. Both studies were reported as a conference abstract only. Egami *et al*.^39^ aimed to predict very late recurrence. Jarman *et al*.^38^ included only patients with persistent AF. Recurrence rates were 21% in the development cohort in Egami *et al*.^39^ and not reported for the other cohorts. Both studies found an association between higher risk scores and recurrence but did not report model performance.

#### Studies comparing models

**Figure 4:**
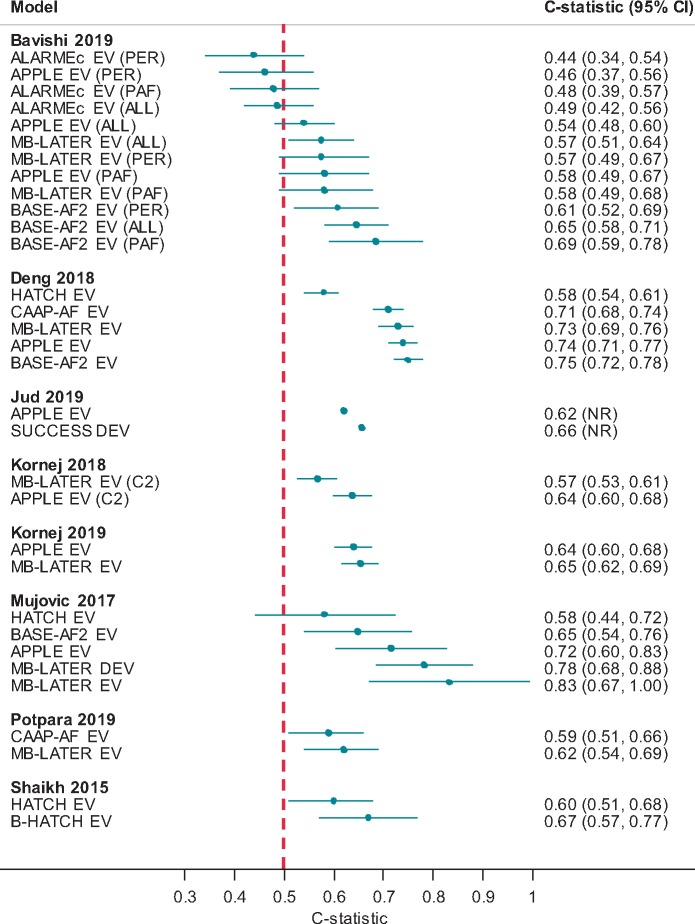
Model c-statistics (by study). ALL, all patients; C1, cohort 1; C2, cohort 2; CI, confidence interval; DEV, model development; EV, external model validation; PAF, paroxysmal AF sub-group; PER, persistent AF sub-group.

Eight studies^36^^,^^37^^,^^44^^,^^46^^,^^55^^,^^64^^,^^66^^,^^67^ compared two or more risk scores in the same population. There was no consistency across studies in terms of which models were compared, and no model consistently showed better discrimination based on the c-statistic (*Figure 4*).

Four studies^37^^,^^46^^,^^64^^,^^66^ reported risk reclassification measures such as NRI or IDI, albeit without CIs, and/or undertook decision curve analysis (Supplementary material online, *File S6*). Findings suggested that (i) adding BNP as a variable (to HATCH) may improve the model,^46^ (ii) MB-LATER may be able to better predict recurrence compared with APPLE, ALARMEc, BASE-AF_2_, and HATCH,^37^ (iii) MB-LATER, BASE-AF_2_, APPLE, and CAAP-AF showed similar clinical usefulness but are more useful than HATCH,^66^ and (iv) MB-LATER showed greater clinical usefulness compared with CAAP-AF.^64^

## Discussion

### Main findings

This systematic review found 33 studies developing and/or validating 13 models to predict AF recurrence after ablation. Model discriminatory ability based on the c-statistic was reported for around 60% of analyses and was highly variable—from very poor to very good. No model had consistently poor or good discriminatory ability across studies. Eight studies compared two or more models in the same population, again with no model showing consistently better discrimination compared with others.

Model calibration was only reported by two studies, and assessment of overall model performance therefore remains incomplete. While our systematic review suggests that clinical risk prediction of recurrent AF after ablation has potential, there is a need for robust evaluation of risk factors and development of risk scores.

The most common model variables were left atrial parameters, type of AF and age, and to a lesser extent sex and eGFR. All model variables can be measured before ablation and therefore models could be used pre-procedurally to predict the likelihood of recurrence. The exception are those models (MB-LATER, BASE-AF_2_, and FER2CI) including early recurrence (within 3 months after ablation) as a variable; these scores can hence only be used to predict late recurrence. Given the inconsistent and sometimes poor performance of the models to date, it is possible that incorporating other variables may improve model performance. There may be a role for biomarkers in assessing AF risk, including serum biomarkers such as BNP^70^^,^^71^ or fibroblast growth factor 23,^70^ imaging of atrial function, ECG-based parameters, and genetic factors.^19^ Some as yet unvalidated models (Supplementary material online, *File S3*) include additional variables. A large ongoing study from South Korea (NCT02138695) plans to develop a simulation model to predict recurrence based on clinical, electrophysiological, anatomical, imaging, and serological characteristics. Clearly, these efforts would benefit from robust evaluation of clinical candidate predictors for recurrent AF after ablation.

### Issues identified

A major risk of bias is that none of the development studies performed internal validation, which may result in overly optimistic and/or biased model performance estimates. This is reflected in *Figure 3*, which shows that c-statistics reported for development studies are often higher than those of validation studies. Overestimation of model performance is more likely to occur when the number of events per candidate predictor is low, model variables are dichotomized based on study data, variables are selected by univariate analyses and weights are incorrectly assigned to predictors. These were all commonly encountered issues. Whilst external validation studies mostly applied the models as originally developed and thus met this quality criterion, this does not mitigate the fact that models were often poorly developed in the first place. Furthermore, around half of studies undertaking external validation did not have a sufficiently large event rate to minimize bias in effect estimates. Risk of bias assessment was hampered by poor reporting, especially on completeness and handling of missing data, as well as predictor assessment. Poor reporting was not limited to conference abstracts but also seen across full-text studies; this is a recognized issue in prognostic research, despite the existence of reporting guidelines.^72^ For comparisons of models, we note that interpretation of both the NRI and the IDI are considered problematic in terms of magnitude and clinical applicability and thus any inferences regarding superior model performance should be regarded as uncertain.^73^

In addition to risk of bias, we also considered the GRADE criteria of imprecision, inconsistency, indirectness, and publication bias. There were concerns regarding indirectness as some models were not applied in the population they were developed in, or for the purpose they were developed for. So for example, HATCH was developed to predict progression to persistent AF but is commonly used to predict AF recurrence after ablation. MB-LATER was developed to predict very late recurrence (>12 months post-ablation) but has been applied in studies to predict recurrence after 3 months. In terms of precision, CIs around c-statistics were often wide, and many encompassed values that spanned weak to good model performance; seven (33%) studies reporting a c-statistic did not report a CI. Heterogeneity could not be quantified since we did not perform a meta-analysis, but inconsistency in discriminatory ability is evident within groups of studies for individual models. Variability may stem from differences in populations, ablation procedure, length of follow-up, and intensity of outcome ascertainment. Publication bias was not assessed as no meta-analysis was performed; it is however known to be an issue in prognosis research.^74^

### Strengths of review and future directions

This systematic review used sensitive search strategies and identified more studies than reported in previous reviews. To the best of our knowledge, it is also the first systematic review in this area to conduct detailed risk of bias assessment using PROBAST. Whilst heterogeneity precluded meta-analysis, results have been presented where possible in forest plots. Screening of all references was performed by only one reviewer due to the large number of references retrieved; the potential for missed studies was mitigated by reference checking of relevant reviews and primary studies, searching in conference abstract databases and screening of a sub-set of references by more than one reviewer.

Impact studies quantify the effect of using a model on decision-making and patient outcome. No studies were identified that looked at the impact of using risk categories based on model scores to influence clinical practice. Given the performance of the models to date, an impact study would likely be premature. Equally, a focus on developing ever more models may not be helpful unless these are more rigorously developed or validated. Future research could focus on revalidating existing models using more methodologically sound approaches particularly with regard to internal validation, variable selection and weighting, assessment of model calibration, and reporting of methods used. Future model development and validation studies may also want to consider pre-specifying sub-groups, e.g. patients with persistent and paroxysmal AF, or first or repeat ablation. Prospective measurement of model variables and outcomes would ensure that patients are not selected based on availability of variable or outcome data, whilst continuous assessment of outcome using implanted devices would be more effective for detecting the outcome. It is recognized that AF is caused by different mechanisms which are currently not targeted by treatment strategies.^75^^,^^76^ Research is ongoing to identify clinical markers related to potential causal mechanisms and to integrate these into prediction models; this may ultimately allow development of more tailored approaches to prevention and therapy.^76^ Future research on model development and validation will likely need to consider differences in underlying causal mechanisms to ensure that models are an appropriate fit to different patient groups.

## Conclusions

Whilst our systematic review suggests that clinical risk prediction of recurrent AF after ablation has potential, there is a need for robust evaluation of risk factors and further development of risk scores to achieve clinical utility.

## Supplementary Material

euaa041_Supplementary_Data
