# Supplementary material for: Predicting recurrent atrial fibrillation after catheter ablation: a systematic review of prognostic models
Source: Europace. 2020 Mar 30;22(5):748–60. doi: 10.1093/europace/euaa041 (PMC7203634; doi:10.1093/europace/euaa041)
Supplement: euaa041_Supplementary_Data [file euaa041_supplementary_data.zip › euaa041-suppl_data/Suppl file 1 Search strategies MEDLINE & Embase.docx]

**Search strategy EMBASE**

1 atrial fibrillation.ti,ab.

2 exp atrial fibrillation/

3 1 OR 2

4 exp recurrent disease/

5 recur$.ti,ab.

6 exp incidence/

7 inciden$.ti,ab.

8 new onset.ti,ab.

9 new$ diagnos$.ti,ab.

10 progress$.ti,ab.

11 prevalen$.ti,ab.

12 exp prevalence/

13 OR/ 4-12

14 prognos$ model$.ti,ab.

15 exp prognosis/ and exp model/

16 predict$ model$.ti,ab.

17 exp prediction/ and exp model/

18 prognos$ index.ti,ab.

19 predict$ index.ti,ab.

20 risk$ predict$.ti,ab.

21 risk$ model$.ti,ab.

22 predict$ rule$.ti,ab.

23 prognos$ rule$.ti,ab.

24 risk score$.ti,ab.

25 clinical score$.ti,ab.

26 prognos$ risk.ti,ab.

27 decision rule$.ti,ab.

28 model$ score$.ti,ab.

29 model performance.ti,ab.

30 decision model.ti,ab.

31 validation.ti,ab.

32 stratification.ti,ab.

33 ROC curve.ti,ab.

34 exp receiver operating characteristic/

35 discriminat$.ti,ab.

36 c-statistic.ti,ab.

37 "Area under the curve".ti,ab.

38 AUC.ti,ab.

39 Calibration.ti,ab.

40 indices.ti,ab.

41 algorithm.ti,ab.

42 multivaria$.mp.

43 (HATCH adj3 scor$).ti,ab.

44 (ALARMEc adj3 scor$).ti,ab.

45 (BASE-AF2 adj3 scor$).ti,ab.

46 (APPLE adj3 scor$).ti,ab.

47 (CAAP-AF adj3 scor$).ti,ab.

48 (MB-LATER adj3 scor$).ti,ab.

49 (CHARGE-AF adj3 scor$).ti,ab.

50 (HAVOC adj3 scor$).ti,ab.

51 (ATLAS adj3 scor$).ti,ab.

52 (FHS adj3 scor$).ti,ab.

53 (ARIC adj3 scor$).ti,ab.

54 (WHS adj3 scor$).ti,ab.

55 (CHADS2 adj3 scor$).ti,ab.

56 (CHA2DS2-VASC adj3 scor$).ti,ab.

57 (R2CHADS2 adj3 scor$).ti,ab.

58 OR/14-57

59 3 AND 13 AND 58

**Search strategy MEDLINE**

1 atrial fibrillation.ti,ab.

2 exp atrial fibrillation/

3 1 or 2

4 exp recurrence/

5 recur$.ti,ab.

6 exp incidence/

7 inciden$.ti,ab.

8 new onset.ti,ab.

9 new$ diagnos$.ti,ab.

10 progress$.ti,ab.

11 prevalen$.ti,ab.

12 exp prevalence/

13 OR/ 4-12

14 prognos$ model$.ti,ab.

15 exp prognosis/ and model.ti,ab.

16 predict$ model$.ti,ab.

17 prognos$ index.ti,ab.

18 predict$ index.ti,ab.

19 risk$ predict$.ti,ab.

20 risk$ model$.ti,ab.

21 predict$ rule$.ti,ab.

22 prognos$ rule$.ti,ab.

23 risk scor$.ti,ab.

24 clinical scor$.ti,ab.

25 prognos$ risk.ti,ab.

26 decision rule$.ti,ab.

27 model$ scor$.ti,ab.

28 model performance.ti,ab.

29 decision model$.ti,ab.

30 validation.ti,ab.

31 (model adj2 validat$).ti,ab.

32 stratification.ti,ab.

33 ROC curve$.ti,ab.

34 exp ROC curve/

35 discriminat$.ti,ab.

36 c-statistic.ti,ab.

37 "Area under the curve".ti,ab.

38 AUC.ti,ab.

39 calibration.ti,ab.

40 indices.ti,ab.

41 algorith$.ti,ab.

42 multivaria$.ti,ab.

43 (HATCH adj3 scor$).ti,ab.

44 (ALARMEc adj3 scor$).ti,ab.

45 (BASE-AF2 adj3 scor$).ti,ab.

46 (APPLE adj3 scor$).ti,ab.

47 (CAAP-AF adj3 scor$).ti,ab.

48 (MB-LATER adj3 scor$).ti,ab.

49 (CHARGE-AF adj3 scor$).ti,ab.

50 (HAVOC adj3 scor$).ti,ab.

51 (ATLAS adj3 scor$).ti,ab.

52 (FHS adj3 scor$).ti,ab.

53 (ARIC adj3 scor$).ti,ab.

54 (WHS adj3 scor$).ti,ab.

55 (CHADS2 adj3 scor$).ti,ab.

56 (CHA2DS2-VASC adj3 scor$).ti,ab.

57 (R2CHADS2 adj3 scor$).ti,ab.

58 OR/14-57

59 3 and 13 and 58

*NB Searches were not restricted to a post-ablation population, as they were developed as part of a wider project.*
