# Supplementary material for: Predicting recurrent atrial fibrillation after catheter ablation: a systematic review of prognostic models
Source: Europace. 2020 Mar 30;22(5):748–60. doi: 10.1093/europace/euaa041 (PMC7203634; doi:10.1093/europace/euaa041)
Supplement: euaa041_Supplementary_Data [file euaa041_supplementary_data.zip › euaa041-suppl_data/Suppl file 2 Risk of bias assessment.docx]

**RISK OF BIAS ASSESSMENT TABLES AND KEY FOR RISK OF BIAS ASSESSMENT**

**Risk of bias assessment: population, predictors and outcomes (based on PROBAST)**

| **Study** | **Model** | | **Appropriate data source?** | **In/exclusions of participants appropriate?** | **Predictors defined and assessed in similar way for all participants?** | **Predictor assessment made without knowledge of outcome data?** | **All predictors available at the time the model is intended to be used?** | **Outcome determined appropriately?** | **Pre-defined/standard outcome definition?** | **Predictors excluded from the outcome definition?** | **Outcome defined and determined in a similar way for all participants?** | **Outcome determined without knowledge of predictor information?** | **Appropriate time interval between predictor and outcome assessment?** |
| --- | --- | --- | --- | --- | --- | --- | --- | --- | --- | --- | --- | --- | --- |
| Berkowitsch 2012 | **“Risk score”** | **DEV √**  INT VAL X  EXT VAL X  U/M X  COMP X | PY | Consecutive patients included; no details on eligibility or exclusion criteria. | PY | PY | Y | Y | Y | Y | PY | NI | Y |
| Wojcik 2013 | **ALARMEc** | **DEV √**  INT VAL X  EXT VAL X  **U/M √**  COMP X | PY | Exclusion criteria: acute reversible causes for AF, valvular AF, known bleeding diathesis, intolerance of heparin or oral  anticoagulation, left atrial thrombus, pregnancy  or breastfeeding, abuse of drugs or alcohol, New  York Heart Association class IV, other severe comorbidity. | PY | Y | Y | Y | Y | Y | PY | NI | PY  (Y for most  N for few -exact numbers not stated; minimum 6 months FU) |
| Wojcik 2014 | **ALARMEc** | DEV X  INT VAL X  **EXT VAL √**  **U/M √**  COMP X | PY | Exclusion criteria defined as study above (except no mention of valvular AF). | PY | PY | Y | Y | PY | Y | PY | NI | Y |
| Wojcik 2015 | **ALARMEc** | DEV X  INT VAL X  **EXT VAL √**  U/M X  COMP X | PY | Exclusion criteria: reversible cause of AF, valvular stenosis or insufficiency, previ­ous MI, congenital heart disease, LVEF < 50%, CABG within the last 3 months, COPD treated with beta-sympathomimetic drugs, severe respiratory insufficiency, known bleeding diathesis, intolerance of heparin or oral anticoagulation, at­tempted AF ablation in the past, left atrial throm­bus, pregnancy or breastfeeding, New York Heart Association class IV, severe comorbidity, abuse of drugs of alcohol, diameter of any PV or common ostium > 27 mm in MRI, no signed informed consent. | PY | PY | Y | Y | Y | Y | PY | NI | Y |
| Kornej 2015  Cohort 1 | **APPLE** | DEV X  INT VAL X  **EXT VAL √**  U/M X  COMP X | NEI  No details on whether all/ consecutive patients. | NI | PY | PY | Y | Y | Y | Y | PY | NI | Y |
| Kornej 2015  Cohort 2 |  |  | NEI  No details on whether all/ consecutive patients. | NI | PY | PY | Y | Y | Y | Y | PY | NI | Y |
| Furukawa 2016  ***Conf abstract***  DEV (“risk score”) | **“Risk score”, APPLE**  *NB Risk score not further considered as not validated* | **DEV √**  INT VAL X  **EXT VAL √**  U/M X  COMP X | PY | NI | PY | PY | Y | NI | Y | Y | PY | NI | PN for a proportion |
| VAL (APPLE) |  |  | PY | NI | PY | PY | Y | NI | Y | Y | PY | NI | PN for a proportion |
| Kornej 2017 | **APPLE** | DEV X  INT VAL X  **EXT VAL √**  U/M X  COMP X | PY | NI | PY | Y | Y | Y | Y | Y | PY | NI | Y |
| Kornej 2018  COHORT 1 | **APPLE, MB-LATER, DR-FLASH** | DEV X  INT VAL X  **EXT VAL √**  U/M X  **COMP √** | PY | Exclusion criteria: pregnancy,  age <18 or >75, valvular AF, cancer, acute or systemic inflammatory diseases. Patients included on basis of available variable data. | PY | PY | Y* | Y | Y | Y | PY | NI | Y |
| Kornej 2018  COHORT 2 |  |  | PY |  | PY | PY | Y* | Y | Y | Y | PY | NI | Y |
| Miake 2018 | **APPLE** | DEV X  INT VAL X  **EXT VAL √**  U/M X  COMP X | Y | Exclusion criteria: age < 20 years or > 85 years, cerebral or myocardial infarction within 6 months of onset, uncontrolled heart failure or LVEF< 40%; severe COPD, contraindications to anticoagulants, thrombus formation within the left atrial appendage, LAD > 60 mm, valvular insufficiency or stenosis, connective tissue disease, active cancer, hypertrophic/dilated cardiomyopathy, old myocardial infarction, end-stage renal disease or haemodialysis. | PY | Y | Y | Y | Y | Y | PY | NI | Y |
| Jud 2019 | **APPLE, SUCCESS** | **DEV √**  INT VAL X  **EXT VAL √**  **U/M √**  **COMP √** | PY | Appears that all patients from one centre included. No mention of exclusions. | PY | NEI | Y | Y | Y | Y | PY | NI | PY  (Y for 74%; PN for 26%). |
| Kornej 2019 | **APPLE, MB-LATER** | DEV X  INT VAL X  **EXT VAL √**  U/M X  **COMP √** | PY | NI | PY | Y | Y* | Y | Y | Y | PY | NI | Y |
| Mesquita 2018  DEV | **ATLAS** | **DEV √**  **INT VAL √**  EXT VAL X  U/M X  COMP X | PY | Excluded those without quantification of LAV data; no details on similarity of clinical characteristics of those included and excluded. | N | PY | Y | Y | Y | Y | PY | NI | Y |
| Mesquita 2018  VAL |  |  | PY |  | N | PY | Y | Y | Y | Y | PY | NI | Y |
| Canpolat 2013 | **BASE-AF_2_** | **DEV √**  INT VAL X  EXT VAL X  U/M X  COMP X | Y | Patients excluded with: moderate to severe valvular disease, thrombus in left atrium, uncontrolled thyroid dysfunction, significant coronary stenosis, contraindication of anticoagulation, pregnancy and LAD >55mm. | PY | Y | Y* | Y | Y | Y | PY | NI | Y |
| Winkle 2016  DEV | **CAAP-AF** | **DEV √**  INT VAL X  **EXT VAL √**  U/M X  COMP X | PY | NI | PY | Y | Y | Y | Y | Y | PY | NI | PY  (Mean 2.5 (1.7) yrs) |
| Winkle 2016  VAL |  |  | Y | NI | PY | Y | Y | Y | Y | Y | PY | NI | PY  (Mean 1.8 (0.9) yrs) |
| Sanhoury 2017 | **CAAP-AF** | DEV X  INT VAL X  **EXT VAL √**  U/M X  COMP X | NEI | NI | PY | PY | Y | Y | Y | Y | PY | NI | Y |
| de Vos 2010  Aim is not to predict recurrence post-ablation, but progression from PAF to persistent AF. Not a post-ablation population, but score is used in post-ablation populations. | **HATCH** | **DEV √**  INT VAL X  EXT VAL X  U/M X  COMP X | PN  Consecutive patients from several centres, but analysis based on sub-group only. | Only included PAF with spontaneous or pharmacological restoration of sinus rhythm. Excluded patients who underwent electrical cardioversion to sinus rhythm and persistent AF. | PY | PY | Y | NI | PY (NB not recurrence but progression) | Y | PY | NI | Y |
| Tang 2010  ***Conference abstract*** | **HATCH** | DEV X  INT VAL X  **EXT VAL *√***  U/M X  COMP X | PY | NI | NEI | NI | Y | NI | NI | Y | NI | NI | NEI  (Mean 474 (330) days; PN for some) |
| Tang 2012 | **HATCH** | DEV X  INT VAL X  **EXT VAL *√***  U/M X  COMP X | PY | NI | PY | PY | Y | Y | Y | Y | PY | NI | PY  (Y (for most)  PN for a small proportion) |
| Silva 2011  ***Conference abstract*** | **HATCH** | DEV X  INT VAL X  **EXT VAL *√***  U/M X  COMP X | NI | NI | PY | Y | Y | NEI | NI | Y | NI | NI | Y |
| Schmidt 2014 | **HATCH** | DEV X  INT VAL X  **EXT VAL *√***  U/M X  COMP X | PY | Patients who had undergone previous catheter ablation or intraoperative ablation for AF were ex­cluded. | PY | Y | Y | Y | Y | Y | PY | NI | NEI  (Follow-up 12.7 (7.1) after mean of 1.38 procedures) |
| Miao 2012  *(part translated from Chinese)* | **HATCH** | DEV X  INT VAL X  **EXT VAL *√***  U/M X  COMP X | PY | NI | PY | NI | Y | PY | Y | Y | PY | NI | Y |
| Shaikh 2014  ***Conference abstract*** | **HATCH** | DEV X  INT VAL X  **EXT VAL *√***  **U/M *√***  COMP X | NI | NI | NI | NI | Y | NEI | NI | Y | NI | NI | N  6 months |
| Shaikh 2015 | **HATCH+OSA**  **B-HATCH** | DEV X  INT VAL X  **EXT VAL *√***  **U/M *√***  COMP X | PY | Patients excluded if history of prior AF ablation. | PY | PY | Y | Y | Y | Y | PY | N | N |
| Chen 2015 | **HATCH** | DEV X  INT VAL X  **EXT VAL *√***  U/M X  COMP X | PY | Exclusion criteria: age<18, contraindication to anticoagulation, prior non-pharmacological interventions for atrial flutter and prior documentation of AF. | PY | NI | Y | Y | Y | Y | PY | NI | PN |
| Garcia-Seara 2016 | **HATCH** | DEV X  INT VAL X  **EXT VAL *√***  U/M X  COMP X | PY | Exclusion criteria: patients with intra-atrial re-entrant tachycardia following reparative surgery for complex congenital heart disease were excluded, patients who underwent concomitant AF ablation during the same  procedure. | PY | PY | Y | Y | Y | Y | PY | NI | Y |
| Providencia 2017  ***Conference abstract*** | **HATCH, AF-FREEDOM**  *NB AF FREEDOM not further considered as not validated* | **DEV *√***  INT VAL X  **EXT VAL *√***  U/M X  **COMP *√*** | NEI | NI | PY | NI | Y | NI | NI | Y | PY | NI | PN |
| Mujovic 2017  DEV | **MB-LATER, APPLE, ALARMc, BASE-AF_2_, HATCH** | **DEV *√***  INT VAL X  **EXT VAL *√***  U/M X  **COMP *√*** | PY | Eligibility based on length of follow-up and being AF free for 12 months; no additional information. | PY | Y | Y* | Y | Y | Y | PY | NI | Y |
| Mujovic 2017  VAL |  |  | PY |  | PY | Y | Y* | Y | Y | Y | PY | NI | Y |
| Potpara 2019 | **MB-LATER, CAAP-AF** | DEV X  INT VAL X  **EXT VAL √**  U/M X  **COMP √** | PY | Eligibility based on ≥12 month follow-up. | PY | PY | Y* | Y | Y | Y | PY | NI | Y |
| Kaplan 2018  ***Conference abstract*** | **MB-LATER** | DEV X  INT VAL X  **EXT VAL √**  U/M X  COMP X | NEI | Eligibility based on being AF free for 12 months; no additional information. | PY | NI | Y* | NI | NI | Y | PY | NI | PY |
| Deng 2018 | **MB-LATER, APPLE, HATCH, BASE-AF_2_, CAAP-AF** | DEV X  INT VAL X  **EXT VAL √**  U/M X  **COMP √** | PY | NI | PY | PY | Y* | Y | Y | Y | PY | NI | Y |
| Bavishi 2019 | **MB-LATER, APPLE, ALARMEc, BASE-AF2** | DEV X  INT VAL X  **EXT VAL √**  U/M X  **COMP √** | PY | Patients who had concomitant cavotricuspid isthmus ablation were excluded. | PY (except potentially LAD) | PY | Y* | Y | Y | Y | PY | NI | Y |
| Jarman 2012  ***Conf abstract***  Cohort 1 DEV | **“Simple score”** | **DEV √**  INT VAL X  **EXT VAL √**  U/M X  COMP X | NEI | NI | PY | NI | Y | NI | Y | Y | PY | NI | PN |
| Jarman 2012  ***Conf abstract***  Cohort 2 VAL |  |  | NEI | NI | PY | Y | Y | NI | Y | Y | PY | NI | PN |
| Egami 2017 ***Conf abstract***  Cohort 1 DEV | **FER2CI score** | **DEV √**  INT VAL X  **EXT VAL √**  U/M X  COMP X | PY | NI | NI | NI | Y* | NI | NI | Y | NI | NI | PY |
| Egami 2017 ***Conf abstract***  Cohort 2 VAL |  |  | NI | NI | NI | NI | Y* | NI | NI | Y | NI | NI | NEI |

CABG = coronary artery bypass graft; COMP=study which compares two or more models; DEV=model development study; EXT VAL =study with external validation of a model; INT VAL= study with internal model validation; LAD=left atrial diameter; LAV=left atrial volume; LVEF=left ventricular ejection fraction; N=no; NEI=not enough information; NI=no information; PN=probably no; PY=probably yes; U/M =study which updates or modifies a model; Y=yes.

* relates to MB-LATER, BASE-AF_2_ and FER2CI scores which include early recurrence as a variable for prediction of late recurrence, so score cannot be used pre-procedurally

**Risk of bias assessment: analysis (based on PROBAST)**

| **Study** | **Model** | **Was there a reasonable number of participants with the outcome?** | **Were continuous and categorical predictors handled appropriately? For validation: was model evaluated as originally fitted?** | **Were all enrolled participants included in the analysis?** | **Were participants with missing data handled appropriately?** | **Was selection of predictors based on univariate analysis avoided? (DEV only)** | **Were complexities in the data (e.g. censoring, competing risks, sampling of control participants) accounted for appropriately?*** | **Were relevant model performance measures evaluated appropriately?** | **Were model overfitting and optimism in model performance accounted for? (DEV only)** | **Do predictors and their assigned weights in the final model correspond to the results from the reported multivariable analysis? (DEV only)** | **Where applicable: Appropriate quantification of added value (0ne score compared to another)? Appropriate method of updating model?** |
| --- | --- | --- | --- | --- | --- | --- | --- | --- | --- | --- | --- |
| Berkowitsch 2012 | **“Risk score”**  **DEV √**  INT VAL X  EXT VAL X  U/M X  COMP X | PY  23 candidate variables; 332 events. 14 EPV. | N  Continuous variables dichotomised based on ROC curve analysis of data. | NI | NI | N  Based on univariate analysis | Y  Cox model accounts for censoring. | N  No calibration measures reported. | N  No valid method for internal validation. | NI  No information on weights assigned. | N/A |
| Wojcik 2013 | **ALARMEc**  **DEV √**  INT VAL X  EXT VAL X  **U/M √**  COMP X | PN  Unclear how many candidate variables for amending model. 88 events. | N  NB One variable (cardiomyopathy) added to “risk score” as above (Berkowitsch 2012) and one variable cut-off (NLA) changed. | Y  All consecutive patients  undergoing CA included for analysis. | N/A | NI | Y  Cox model accounts for censoring. | N  No calibration measures reported. | N  No internal validation. | NI | N  No details on model updating/  modification. |
| Wojcik 2014 | **ALARMEc**  DEV X  INT VAL X  **EXT VAL √**  **U/M √**  COMP X | Y  219 events | N  Cut-off for NLA used as in Berkowitsch 2012 so in effect a new model. | Y  States that all consecutive patients undergoing CA were included in analysis. | N/A |  | N/A  No model re-fitting. | N  No discrimination or calibration measures reported. |  |  | N  No details on model updating/  Modification. |
| Wojcik 2015 | **ALARMEc**  DEV X  INT VAL X  **EXT VAL √**  U/M X  COMP X | Y  125 events | PN  Model variables/cut-offs changed over time. | Y  States that all consecutive patients undergoing CA were included in analysis. | N/A |  | N/A  No model re-fitting | N  No discrimination or calibration measures reported. |  |  | N/A |
| Kornej 2014  Variables for development of APPLE score taken from this study; however, this study did not include development of APPLE score. No further development study for this score was identified. | | | | | | | | | | | |
| Kornej 2015  Cohort 1 | **APPLE**  DEV X  INT VAL X  **EXT VAL √**  U/M X  COMP X | Y  379 events | NI  No information on how variable cut-offs chosen; not a development study but first time score is defined and used. | NI | NI |  | N/A  No model re-fitting | N  No calibration measures reported. |  |  | N/A |
| Kornej 2015  Cohort 2 |  | Y  185 events | Y  Appears score applied as in VAL 1 cohort. | NI | NI |  |  |  |  |  |  |
| Furukawa 2016  ***Conf abstract***  DEV (“risk score”) | **“Risk score”, APPLE**  **DEV √**  INT VAL X  **EXT VAL √**  U/M X  COMP X | NI  Number of candidate predictors not known. | N  Variable cut-offs based on ROC analysis of data. | NI | NI |  | Y  Cox analysis. | N  No calibration measures reported. | PN  No details on any form of internal validation | NI  No details on how points were assigned. | N  Difference (p-value) calculated for AUCs.  NB Only APPLE considered as risk score not validated |
| VAL (APPLE) |  | Y  150 events | PY  Assume APPLE score applied as previously defined. | NI | NI |  | N/A  No model re-fitting | N  No calibration measures reported. |  |  |  |
| Kornej 2017 | DEV X  INT VAL X  **EXT VAL √**  U/M X  COMP X | Y  133 events (NB includes atrial tachycardia) | Y  Appears score applied as in Kornej 2015. | NI | NI |  | N/A  No model re-fitting | N  No calibration measures reported. |  |  | N/A |
| Kornej 2018  COHORT 1 | **APPLE, MB-LATER, DR-FLASH**  DEV X  INT VAL X  **EXT VAL √**  U/M X  **COMP √** | N  64 events | Y  Score applied as previously defined. | NI  NB Patients included on basis of available variable data. | NI |  | N/A  No model re-fitting | N  No calibration measures |  |  | No measures that quantify added benefit of using one score over another. |
| Kornej 2018  COHORT 2 |  | Y  300 events |  |  |  |  |  |  |  |  |  |
| Miake 2018 | **APPLE**  DEV X  INT VAL X  **EXT VAL √**  U/M X  COMP X | N  65 events. | Y  Score applied as previously defined. | NI | NI |  | N/A  No model re-fitting | N  No discrimination or calibration measures |  |  | N/A |
| Jud 2019  DEV (SUCCESS) | **SUCCESS, APPLE**  **DEV √**  INT VAL X  **EXT VAL √**  **U/M √**  **COMP √** | NI for DEV  (no information on candidate predictors) | Y  APPLE score used as previously developed. | NEI  Appears that all patients from one centre included. No mention of exclusions. | NI | N  Included variables identified as significant on univariate analysis. | N  Censoring not considered in model development. | Y  (NB assessment of calibration based on Hosmer-Lemeshow test which has limited power). | N  No internal validation of newly created SUCCESS score. | NI  No information on weights assigned. | No attempt to quantify added value of SUCCESS score. |
| Jud 2019  EXT VAL  (APPLE) |  | N  32 events | N/A  SUCCESS score includes additional point for each previous ablation performed; no additional continuous variables. |  |  |  | N/A  No model refitting |  |  |  |  |
| Kornej 2019 | **APPLE, MB-LATER**  DEV X  INT VAL X  **EXT VAL √**  U/M X  **COMP √** | Y  562 events | Y  Scores applied as previously defined. | NI | NI |  | N/A  No model re-fitting | N  No calibration measures |  |  | No quantification of differences between scores. |
| Mesquita  2018  DEV | **ATLAS**  **DEV √**  **INT VAL √**  EXT VAL X  U/M X  COMP X | Y  >20 events per candidate variable | PN  Appears that cut-offs based on the study data. | NEI  Patients with missing variable data excluded and 6% lost to FU. No details on characteristics of excluded patients. | N  Patients with missing variable data and lost to FU excluded from analysis. | N  Variables selected on basis of univariate analysis. | Y  Cox model accounts for censoring | Y  c-statistic and calibration measures presented | PN  Split sample inadequate (though reasonable number of events). No mention of bootstrapping or cross-validation. | PY  Score scale based on regression co-efficients. | N/A |
| Mesquita 2018  INT VAL |  | Y  239 events | Y  Score applied as developed. |  |  |  |  |  |  |  |  |
| Canpolat 2013 | **BASE-AF**  **DEV √**  INT VAL X  EXT VAL X  U/M X  COMP X | N  15 candidate variables; 60 events. 4 EPV. | N  Variable cut-offs based on the study data. | NI | NI | N  Variables selected on basis of univariate analysis. | Y  Cox model accounts for censoring | N  No calibration measures reported. | N  No internal validation. | N  Magnitude not considered when assigning points to variables. | N/A |
| Winkle 2016  DEV | **CAAP-AF**  **DEV √**  INT VAL X  **EXT VAL √**  U/M X  COMP X  NB Cohort separated in time could potentially been viewed as split sample but due to large number of events, decision made to treat as external validation. | Y  14 candidate variables, 302 events; 21 EPV. | N  Variable categories based on study data. | NI | NI | Y  Multivariate Cox regression to identify independent predictors. | Y  Cox model accounts for censoring | N  No calibration measures reported. | N  No internal validation. | N  Point based weighting system developed based on degree of separation of KM curves. | N/A |
| Winkle 2016  VAL |  | Y  196 events | Y  Same scoring system used for VAL cohort. | NI | NI |  | N/A  No model re-fitting | N  No calibration measures reported. |  |  | N/A |
| Sanhoury 2017 | **CAAP-AF**  DEV X  INT VAL X  **EXT VAL √**  U/M X  COMP X | N  25 events | Y  Score applied as developed. | NI | NI |  | N/A  No model re-fitting | N  No calibration measures reported. |  |  | N/A |
| de Vos 2010  Aim is not to predict recurrence post-ablation, but progression from PAF to persistent AF. Not a post-ablation population, but score is used in post-ablation populations. | **HATCH**  **DEV √**  INT VAL X  EXT VAL X  U/M X  COMP X | PN  19 (or more) candidate variables (unclear), 178 events. | N  Cut-off for age based on data analysis. | N  Follow-up data unavailable for 31% of patients. These more often had underlying heart disease and a higher HATCH score. | N | N  Variables selected on basis of univariate analysis. | PN  Logistic regression model was used; similar FU period, no censoring.  But better to use time-to-event analysis + censoring. | N  No calibration measures reported. | N  No details on any form of internal validation. | Y  Regression  coefficients of the final logistic regression model were  used to estimate the contribution of each variable to the risk  estimation of AF progression. | N/A |
| Tang 2010  **Conference abstract** | **HATCH**  DEV X  INT VAL X  **EXT VAL *√***  U/M X  COMP X | Y  225 events | Y  Score applied as developed. | NI | NI |  | N/A  No model re-fitting. | N  No discrimination or calibration measures. |  |  | N/A |
| Tang 2012 | **HATCH**  DEV X  INT VAL X  **EXT VAL *√***  U/M X  COMP X | Y  176 events | Y  Score applied as developed. | Y  5/493 (1%) lost to FU-baseline characteristics did not differ so unlikely to make a difference. | N/A |  | N/A  No model re-fitting. | N  No calibration measures reported. |  |  | N/A |
| Silva 2011  **Conference abstract** | **HATCH**  DEV X  INT VAL X  **EXT VAL *√***  U/M X  COMP X | N  <100 (events not stated but only 47 patients in total) | PY  Appears that score applied as developed. | NI | NI |  | N/A  No model re-fitting. | N  No discrimination or calibration measures. |  |  | N/A |
| Schmidt 2014 | **HATCH**  DEV X  INT VAL X  **EXT VAL *√***  U/M X  COMP X | N  72 events. | PY  Appears that score applied as developed. | NI | NI |  | N/A  No model re-fitting. | N  No discrimination or calibration measures. |  |  | N/A |
| Miao 2012  (part translated from Chinese) | **HATCH**  DEV X  INT VAL X  **EXT VAL *√***  U/M X  COMP X | N  43 events. | Y  Score applied as developed. | NI | NI |  | N/A  No model re-fitting. | N  No discrimination or calibration measures. |  |  | N/A |
| Shaikh 2014  ***Conference abstract*** | **HATCH+OSA**  DEV X/√*  INT VAL X  **EXT VAL *√***  **U/M *√***  COMP X  *New model but not a development study. | N  35 events  No details on candidate variables from which OSA selected. | N  HATCH not applied as developed but modified by including an additional variable-OSA. | NI | NI | NI  No details on how OSA selected. | NI  No details relating to time-to-event analysis. | N  No calibration measures. | NI  No details on any form of internal validation. | NI  No details on how points assigned. | No details on how the modified HATCH score compared to the original HATCH score. |
| Shaikh 2015  (DEV HATCH-B) | **HATCH,**  **B-HATCH**  **DEV √**  INT VAL X  **EXT VAL *√***  **U/M *√***  **COMP √** | NI  Number of candidate variables unclear. | Y  Modified HATCH score includes additional variable-BNP ≥100 pg/dL. Cut-off chosen on basis of prior analyses. Other variables in HATCH scores not changed. | N  22/183 (12%) lost to FU and excluded from analysis. | N  Excluded from analysis | Y  BNP ≥100-cut-off chosen on basis of prior analyses. | PN  Logistic regression model was used;  better to use time-to-event analysis + censoring. | N  No calibration measures. | N  No details on any form of internal validation. | N  Points for a baseline BNP ≥100 pg/dL assigned based on relative risk for prediction of recurrence. | Y  NRI and IDI calculated. |
| Shaikh 2015  VAL (HATCH) |  | N  77 events | Y  Score applied as developed (HATCH). |  |  |  | N/A  No re-fitting of model |  |  |  |  |
| Chen 2015 | **HATCH**  DEV X  INT VAL X  **EXT VAL *√***  U/M X  COMP X | N  85 events | Y  Score applied as developed. | Y  17/233 (7%) excluded (lack of follow-up or AFL recurrence)- stated that similar characteristics so unlikely to make a difference | N/A |  | N/A  No re-fitting of model | N  No calibration measures. |  |  | N/A |
| Garcia-Seara 2016 | **HATCH**  DEV X  INT VAL X  **EXT VAL *√***  U/M X  COMP X | N  75 events (recurrence) | Y  Score applied as developed. | Y  States that all admitted patients were included. | N/A |  | N/A  No re-fitting of model | N  No discrimination or calibration measures. |  |  | N/A |
| Providencia 2017  ***Conference abstract***  DEV | **HATCH, AF-FREEDOM**  **DEV *√***  INT VAL X  **EXT VAL *√***  U/M X  **COMP *√*** | NI  No details on number of candidate variables (AF-FREEDOM) | N  Variable cut-offs based on data | NI | NI | Y  Independent predictors identified through multivariate analysis. | Y  Cox regression. | N  No calibration measures. | NI | PY  Assignment of points to predictors related to co-efficients. | No measures for comparing the models.  AF-FREEDOM not further considered as not validated |
| Providencia 2017  ***Conference abstract***  VAL |  | PY  Large sample size (n=1293) | Y  Score applied as developed. (HATCH). |  |  |  | N/A  No re-fitting of model |  |  |  |  |
| Mujovic 2017  DEV  (Cohort 1 MB-LATER) | **MB-LATER, APPLE, ALARMc, BASE-AF2, HATCH**  **DEV *√***  INT VAL X  **EXT VAL *√***  U/M X  **COMP *√*** | N  39 candidate variables and 20 events. | N  Variable cut-offs on basis of ROC curve analysis. | Y  Appears all eligible included (NB eligibility based on length of FU and being AF free for 12 months;  characteristics of included and excluded patients similar) | N/A | N  Variables chosen on basis of univariate analysis. | Y  Cox analysis. | N  No calibration measures. | N  No details of any form of internal validation. | NI | Y  NRI, IDI and decision curve analysis for estimating improvement in predictive ability. |
| Mujovic 2017  VAL  (Cohort 1 other scores)) |  | N  20 events | Y  Scores applied as developed. |  |  |  | N/A  No re-fitting of model |  |  |  |  |
| Mujovic 2017  VAL  (Cohort 2 MB-LATER) |  | N  3 events (based on 39 patients). Not a useful validation-better to have included patients into development cohort. | Y  Score applied as developed. | Y  Appears to be all patients now eligible due to reaching specified FU. | N/A |  | N/A  No re-fitting of model |  |  |  |  |
| Potpara 2019 | **MB-LATER, CAAP-AF**  DEV X  INT VAL X  **EXT VAL √**  U/M X  **COMP √** | Y  133 events | Y  Scores applied as developed. | NEI  Appears to be all patients for whom ≥1 year follow-up data were available (eligibility criterion). | NI |  | N/A  No re-fitting of model | N  No calibration measures. |  |  | Y  Decision curve analysis |
| Kaplan 2018  ***Conference abstract*** | **MB-LATER**  DEV X  INT VAL X  **EXT VAL √**  U/M X  COMP X | N  Number of events not stated but only 96 patients. | Y  Scores applied as developed. | NI | NI |  | N/A  No re-fitting of model | N  No discrimination or calibration measures. |  |  | N/A |
| Deng 2018 | **MB-LATER, APPLE, HATCH, BASE-AF_2_, CAAP-AF**  DEV X  INT VAL X  **EXT VAL √**  U/M X  **COMP √** | Y  365 events | Y  Scores applied as developed. | Y  13/1423 (0.9%) excluded | N/A |  | N/A  No re-fitting of model | N  No calibration measures. |  |  | Y  NDRI, IDI and decision curve analysis for estimating improvement in predictive ability. |
| Bavishi 2019 | **MB-LATER, APPLE, ALARMEc, BASE-AF2**  DEV X  INT VAL X  **EXT VAL √**  U/M X  **COMP √** | Y  147 events. | Y  Scores applied as developed. NB ALARMEc-appears to be based on Wojcik 2013, which has a different NLA cut-off compared with other ALARMEc studies. | NEI  63/674 (9.3%) excluded for having <3 months FU data. | NI |  | N/A  No re-fitting of model | N  No calibration measures. |  |  | N  Several scores evaluated but only p-values compared not added value/improvement. |
| Jarman 2012  ***Conf abstract***  Cohort 1 DEV | **“Simple score”**  **DEV √**  INT VAL X  **EXT VAL √**  U/M X  COMP X | NI  23 candidate variables; number of events not known. | PN  3 categories for LAD, unclear how derived | NI | NI | Y  Multivariate analysis to identify variables | PN  No details on Cox analysis | N  No discrimination or calibration measures. | PN  No details on any form of internal validation | NI | N/A |
| Jarman 2012  ***Conf abstract***  Cohort 2 VAL |  | NI  Number of events not stated. | Y  Score applied as developed | NI | NI |  | N/A  No model re-fitting | N  No discrimination or calibration measures. |  |  | N/A |
| Egami 2017 ***Conf abstract***  Cohort 1 DEV | **FER2CI score**  **DEV √**  INT VAL X  **EXT VAL √**  U/M X  COMP X | PN  Number of candidate predictors not known, but small number of events (n=24) | N  Cut-off for APC based on study data. | NI | NI | NI | NI | N  No discrimination or calibration measures. | N  No details on any form of internal validation. | NI | N/A |
| Egami 2017 ***Conf abstract***  Cohort 2  VAL |  | N  44 events | PY  Assume score applied as developed. | NI | NI |  | N/A  No model re-fitting |  |  |  | N/A |

APC=atrial premature contraction; BNP=brain natriuretic peptide; CA=catheter ablation; COMP=study which compares two or more models; DEV=model development study; EPV=events per variable; EXT VAL =study with external validation of a model; IDI= integrated discrimination index; INT VAL= study with internal model validation; LAD=left atrial dimension; N=no; NEI=not enough information; NI=no information; NLA=normalised left atrial area; NRI=net reclassification index; OSA=obstructive sleep apnoea; PN=probably no; PY=probably yes; U/M =study which updates or modifies a model; Y=yes.

**Risk of bias assessment –key (informed by PROBAST)**

| *Appropriate data source?* | Y if prospective cohort with consecutive patients or all patients admitted during specified time period.  PY if prospective cohort (no further details) or retrospective analysis with consecutive patients (or all patients admitted during specified time period).  NEI if retrospective analysis with no further details or if single centre but with no further information.  PN if data based on a sub-group from a larger patient sample. |
| --- | --- |
| *Were all inclusions and exclusion appropriate?* | No decision made on whether these were appropriate due to (i) poor reporting of the information and (ii) inconsistency in whether eligibility criteria were reported for ablation and/or inclusion into the model analysis. Details, where reported, have been included in the table. |
| *Applicability* –do participants/setting match the review question? | Not included in table. Whilst the vast majority of studies match the review question (i.e. recurrence in post-ablation population), a number of studies were included where there was a discrepancy (for example where the model was developed in a different population, but subsequently applied in a post-ablation population). These studies have been highlighted when considering ‘indirectness’ (GRADE criterion) in the discussion. |
| *Predictors defined and assessed in similar way for all participants?* | Y if reference made to standard criteria used in all patients.  PY if single centre. For some criteria there is a standard way of measuring (e.g. LVEF), others are not prone to measurement issues (e.g. age, sex, number of AADs failed). Some information is unlikely to be reported, e.g. how co-morbidities were defined.  PY if states that methods performed in accordance with relevant guidelines/regulations.  PY if multicentre but standardised protocol.  NI if not able to tell if single centre and no other information.  N if specific statement that variables were measured in different ways. |
| *Predictor assessment made without knowledge of outcome data?* | Y if clear that all score components measured before ablation (and statement to that effect).  PY if appears that (at least some) predictors were measured pre-procedurally (or at admission). There are some standard pre-procedural investigations. Less important for fixed predictors (age, sex).  NI if no details on timing of predictor assessment. |
| *All predictors available at the time the model is intended to be used?* | This is always Y. Only caveat-some models include early recurrence as a predictor for late recurrence-so would not know this before procedure. This has been indicated in the table. |
| *Was the outcome determined appropriately?* | Y if regular follow-up with Holter monitoring and ECG and additional monitoring on symptom recurrence. Some differences between studies in intensity.  PY if ECG and/or Holter mentioned but no details on frequency.  NI if no details-more often in studies reported as abstracts only. |
| *Standard outcome definition?* | Y if AF/left atrial tachycardia recurrence >30 seconds occurring after blanking period of 3 months.  PY if only states clinical and/or electrocardiographic recurrence, or if no details but same study group has previously defined the outcome.  NI if no details-more often in studies reported as abstracts only. |
| *Were predictors excluded from the outcome definition?* | This is always Y. Predictors do not form part of outcome assessment. |
| *Was the outcome defined and determined in a similar way for all participants?* | Y if explicit statement.  PY if the standard definition given and/or single centre centre. |
| *Was the outcome determined without knowledge of predictor information?* | NI- all but one study reported no information regarding this.  N if clear statement that outcome assessment could have been influenced by knowledge of one or more variables. |
| *Appropriate time interval between predictor and outcome assessment?* | Y - if at least 12 months for all.  PY- if likely that at least 12 months for a majority of patients  NI –no (or unclear) information given on length of follow-up  PN –if it is likely that at a majority of patients did not have a 12 month follow-up  N –clearly less than 12 months for all patients.  This was somewhat subjective as sometimes only a mean or median (with or without a range was stated) and the minimum follow-up period for all patients was not known. |
| *Was there a reasonable number of participants with the outcome?* | Development studies  Y -if >20 events per variable for candidate predictors  PY –if > 10 events per variable  NI- no details or unclear how many candidate variables  PN- if number of candidate variables unclear but small sample size  N -<10 events per variable  Validation studies  Y-at least 100 participants with outcome.  PY –if events not stated but very large sample size (e.g. >1000)  NI-number of events not stated  N-less than 100 participants with outcome |
| *Were continuous and categorical predictors handled appropriately?* | Development studies  Y –if no dichotomisation of continuous predictors based on study data or if cut-off predefined (widely accepted) rather than based on the data  PN - appears that one or more cut-offs based on study data  N-if dichotomisation of one or more continuous predictors based on study data (and no adjustment by applying internal validation and shrinkage techniques)  Validation studies  Y- if model being used as originally fitted-same dichotomisation and cut-offs. Using equation/model as created.  PY- appears that same model is being used but not explicit  PN-appears that some model variables have been changed  N-some model variables (or cut-offs) clearly changed |
| *Were all enrolled participants included in the analysis?* | Y- explicit statement that all patients were included in analysis (or if some were excluded –that the characteristics were similar to the included)  NI-no details, or small proportion (<10%) of patients excluded but no information on similarity of patient characteristics between in- and excluded  N-clear statement that >10% patients lost to follow-up (or with missing predictor information) were excluded from the analysis and/or that patient characteristics differed between in-and excluded  Note that sometimes availability of variable or outcome data was as an eligibility criterion. |
| *Were participants with missing data handled appropriately?* | NI-no details on handling of missing data  N- patients lost to follow-up simply excluded (and no details on similarity of patients characteristics between in- and excluded)  N/A in studies where all patients had been included in analysis (or if excluded had similar characteristics to included).  No studies reported an appropriate method for handling missing data, e.g. by using multiple imputation. |
| *Was selection of predictors based on univariate analysis avoided? (DEV only)* | Y- if predictors identified through multivariate analysis  NI –no details on how predictors were selected  N- if predictors selected on the basis of univariable analysis before multivariable modelling |
| *Were complexities in the data (e.g. censoring, competing risks, sampling of control participants) accounted for appropriately?* | No (nested) case control studies included so no need to account for sampling fractions; also assumption made that no substantial competing risks (e.g. death occurring before recurrence).  Development studies  Y- time to event analysis used (e.g. Cox analysis)  NI-no details on type of analysis  PN- logistic regression model used/insufficient information  N-no time-to event analysis used  Validation studies  N/A as none of the validation studies undertook calibration and model refitting. |
| *Were relevant model performance measures evaluated appropriately?* | Y-both a discrimination and calibration statistic reported  N-only one of the above or none presented |
| *Were model overfitting and optimism in model performance accounted for? (DEV only)* | Y-a form of internal validation included (e.g. bootstrapping or cross-validation); where included should adjust or shrink the model predictive performance estimates and predictor effects in the final model  PN- a split sample approach used with >20 events per candidate variable  N-no form of internal validation or a split sample approach with <20 events per candidate variable |
| *Do predictors and their assigned weights in the final model correspond to the results from the reported multivariable analysis? (DEV only)* | Y-regression co-efficients used to estimate contribution of each variable to the risk  PY- score scale based on regression co-efficients (with no further details)  NI-no information on how weights assigned  N-inappropriate method, e.g. assigning points based on relative risk or degree of separation of Kaplan-Meier curves |

DEV=model development study; N=no; NEI=not enough information; NI=no information; PN=probably no; PY=probably yes; Y=yes.
