# Supplementary material for: Predicting recurrent atrial fibrillation after catheter ablation: a systematic review of prognostic models
Source: Europace. 2020 Mar 30;22(5):748–60. doi: 10.1093/europace/euaa041 (PMC7203634; doi:10.1093/europace/euaa041)
Supplement: euaa041_Supplementary_Data [file euaa041_supplementary_data.zip › euaa041-suppl_data/Suppl file 3 Non-validated models.docx]

**Studies not taken forward for data extraction and analysis due to lack of validation or other reasons**

| **Study** | **Model/risk score evaluated** | **Reason for excluding** |
| --- | --- | --- |
| Bieging ET, Morris A, Wilson BD, McGann CJ, Marrouche NF, Cates J. Left atrial shape predicts recurrence after atrial fibrillation catheter ablation. Journal of Cardiovascular Electrophysiology. 2018;29(7):966-72. | Fibrosis and clinical covariates, with and without shape parameters | Internal validation of one or more models conducted but unclear which variables included in final models. |
| Bisbal F, Alarcón F, Ferrero-de-Loma-Osorio A, González-Ferrer JJ, Alonso C, Pachón M, Tizón H, Cabanas-Grandío P, Sanchez M, Benito E, Teis A, Ruiz-Granell R, Pérez-Villacastín J, Viñolas X, Arias MA, Vallés E, García-Campo 9, Fernández-Lozano I, Villuendas R, Mont L. Left atrial geometry and outcome of atrial fibrillation ablation: results from the multicentre LAGO-AF study. Eur Heart J Cardiovasc Imaging. 2018 Sep 1;19(9):1002-1009. | LAGO  (AF phenotype, structural heart disease, CHA2DS2-VASc ≤ 1, LAD, and LA sphericity) | No validation. |
| Choe W, Kang JH, Choi EK, Shin SY, Lim HE, Oh S. Better prediction of response to atrial fibrillation ablation using genetic risk score with common genetic variants. Heart Rhythm. Conference: 38th Annual Scientific Sessions of the Heart Rhythm Society 2017. | Genetic risk score (five single nucleotide polymorphisms) | No validation. |
| Ejima K, Kato K, Arai K, Fukushima K, Henmi R, Yagishita D, et al. Predictive value of atrial remodeling of atrial tachyarrhythmia recurrence after catheter ablation of atrial fibrillation during the long-term follow. Europace. 2015; Conference: EHRA EUROPACE CARDIOSTIM 2015. Milan Italy. Conference Publication: 17 (SUPPL. 3) (pp iii114). | Combination of left atrial volume index and estimated total atrial conduction time | No validation. |
| Faber L, Da Silva DB, Lehmann RB, Prinz C, Horstkotte D. Recurrence of atrial fibrillation in patients assigned to a rhythm control strategy. Journal of the American College of Cardiology.Conference: 63rd Annual Scientific Session of the American College of Cardiology and i2 Summit: Innovation in Intervention. 2014. | Low LAA outflow velocity, large LA size, low EF, advanced age, and absence of catheter ablation | No validation. |
| Furukawa Y, Yamada T, Morita T, Tamaki S, Iwasaki Y, Kawasaki M, et al. A novel and simple risk score for the prediction of recurrence of atrial fibrillation after radio frequency catheter ablation. European Heart Journal. Conference: European Society of Cardiology 2016. | Persistent AF, eGFR and LA diameter | No validation. *NB Study included for its external validation of APPLE score.* |
| He Y, Zhang B, Zhu F, Hu Z, Zhong J, Zhu W. Transesophageal echocardiography measures left atrial appendage volume and function and predicts recurrence of paroxysmal atrial fibrillation after radiofrequency catheter ablation. Echocardiography 35 (7) (pp 985-990), 2018. | Left atrial volume index, left atrial appendage ejection fraction, left atrial appendage peak emptying flow velocity | No validation. |
| Hidalgo-Munoz AR, Latcu DG, Meo M, Meste O, Popescu I, Saoudi N, et al. Spectral and spatiotemporal variability ECG parameters linked to catheter ablation outcome in persistent atrial fibrillation. Computers in Biology & Medicine. 2017; 88:126-31. | ECG parameters (AF dominant frequency, spatiotemporal variability) | No validation. |
| Jongnarangsin K, Suwanagool A, Chugh A, Crawford T, Good E, Pelosi F, Jr., et al. Effect of catheter ablation on progression of paroxysmal atrial fibrillation. Journal of Cardiovascular Electrophysiology. 2012;23(1):9-14. | HATCH | Score used for predicting progression from paroxysmal to persistent AF. |
| Machino T, Tada H, Igarashi M, Sekiguchi Y, Kuroki K, Yamasaki H, et al. A novel scoring system predicts the long-term outcome of catheter ablation in patients with long-standing persistent atrial fibrillation. Heart Rhythm.Conference:35th Annual Scientific Sessions of the Heart Rhythm Society 2014. | Advanced AF duration, failed pharmacologic cardioversion and reduced left atrial appendage flow | No validation. |
| Marrouche NF, Wilber D, Hindricks G, Jais P, Akoum N, Marchlinski F, et al. Association of atrial tissue fibrosis identified by delayed enhancement MRI and atrial fibrillation catheter ablation: the DECAAF study. JAMA 2014;311(5):498-506. | Fibrosis and clinical covariates | No validation. |
| Paylos JM, Morales A, Azcona L, Paradela M, Yague R, Gomez-Guijarro F, et al. Long-Term Evolution of Patients Treated for Paroxysmal Atrial Fibrillation with First and Second Generation Cryoballoon Catheter Ablation with a Prospective Protocol Guided by Complete Bidirectional Left Atrium-Pulmonary Veins Disconnection after Adenosine as Main Target end Point to achieved. Seven Years Follow-up of Patients with a rough estimation profile of Low ALARMEc Score. A Single Center Report. Journal of Atrial Fibrillation. 2016;8(6):1400. | ALARMEc | No individual patient risk scores calculated, only a “rough estimate profile”. |
| Peichl P, Wichterle D, Aldhoon B, Stojadinovic P, Sramko M, Borisincova E, et al. Predictors of good arrhythmia control after catheter ablation for atrial fibrillation. Heart Rhythm.Conference:38th Annual Scientific Sessions of the Heart Rhythm Society 2017. | Persistent AF, female gender, age, left ventricular ejection fraction | No validation. |
| Providencia R, Albenque JP, Combes S, Vieira M, Khoueiry Z, Sousa P, et al. The HATCH score does not predict FREEDOM from Atrial Fibrillation relapse following catheter ablation: Development of a novel stratifying tool. Europace. 2017; Conference: European Heart Rhythm Association EUROPACE-CARDIOSTIM 2017 Congress. Austria. 19 (Supplement 3) (pp iii285). | AF-FREEDOM (persistent AF, longstanding persistent AF, AF at the start of the procedure, severe enlargement of the left atrium, AF duration, morbid obesity, obstructive sleep apnoea and structural heart disease) | No validation. *NB Study included for its external validation of HATCH.* |
