# Supplementary material for: Predicting recurrent atrial fibrillation after catheter ablation: a systematic review of prognostic models
Source: Europace. 2020 Mar 30;22(5):748–60. doi: 10.1093/europace/euaa041 (PMC7203634; doi:10.1093/europace/euaa041)
Supplement: euaa041_Supplementary_Data [file euaa041_supplementary_data.zip › euaa041-suppl_data/Suppl file 4 DR-FLASH score.docx]

**DR-FLASH score**

This score was developed and externally validated by Kosiuk (2015)^1^ to predict low voltage areas (LVAs) in patients who may benefit from extensive substrate modification during catheter ablation. Variables are diabetes, renal dysfunction, type of AF, left atrial diameter, age, sex and hypertension. The score was applied to predict AF recurrence in a separate cohort in the same study; this cohort only included patients who had not undergone substrate modification. The score has subsequently been applied in two cohorts in a further study (Kornej 2018^2^) to predict recurrence of both LVAs and recurrence of AF. Patients across all cohorts were undergoing a first ablation procedure. Follow-up times for recurrence were up to 36 months (Kosiuk 2015^1^) and 12 months (Kornej 2018^2^).

Recurrence rates for AF were between 27% and 34%. A c-statistic (0.66 (95% CI 0.62, 0.70)) was reported for one of the cohorts in Kornej 2018^2^ ; this indicted weak discriminatory ability; odds ratios reported in this study found a statistically significant but small increase in odds for recurrence with increase in risk score for one of the two cohorts only. This association was also found in the Kosiuk 2015^1^ study. Greatest risk of bias stemmed from a lack of internal validation of the score.

**Main study characteristics**

| **Study** | **Model** | **Sample** | **Population characteristics** | **Ablation procedure** | **Antiarrhythmic drugs (AAD)** | **Outcome definition** | **Method of outcome assessment** | | **Length of Follow-up** |
| --- | --- | --- | --- | --- | --- | --- | --- | --- | --- |
| Kosiuk 2015 | **DR-FLASH**  **DEV √**  INT VAL X  **EXT VAL √**  U/M X  COMP X | Development and external validation of the DR-FLASH score to identify left atrial low voltage areas (LVA) and potentially patients who may need extensive substrate modification; also applied to separate cohort for predicting recurrence. | Prospective single centre cohort n=238 Heart Centre Leipzig (DEV) | Age: 61 (10)  Male: 76%  PAF: 37%  Heart failure: 12%  Prior stroke/TIA: NR CAD: NR  Diabetes: 16%  BMI: NR | First ablation. PVI + tailored substrate modification. | Class I and III AADs discontinued after ablation. | High density electroanatomic voltage maps created with ablation catheters or multipolar mapping catheters to characterise the underlying LA substrate. | | N/A |
|  |  |  | Prospective multi-centre cohort n=180 (VAL for LVAs) | Age: 60 (10)  Male: 68%  PAF: 56%  Heart failure: 16%  Prior stroke/TIA: NR CAD: NR  Diabetes: 17%  BMI: NR | First ablation. PVI + tailored substrate modification. |  |  |  | N/A |
|  |  |  | Retrospective cohort of consecutive patients n=484 (VAL for recurrence) | Age: 59 (9)  Male: 68%  PAF: 74%  Heart failure: 6%  Prior stroke/TIA: NR CAD: NR  Diabetes: 13%  BMI: NR | First ablation. PVI. |  | NR | 7-day Holter monitoring performed at 6, 12 and 24 months, then annually. | Up to 36 months. |
| Kornej 2018  COHORT 1  FT | **APPLE, MB-LATER, DR-FLASH**  DEV X  INT VAL X  **EXT VAL √**  U/M X  **COMP √** | Validation of APPLE, MB-LATER and DR-FLASH scores in 2 cohorts. | Retrospective study.  BioAF cohort (Heart Centre Leipzig, Germany)  n=241  Possible overlap with above cohort | Age: Recurrence: 64 (56-69); no recurrence: 65 (58-73)  Male:67%  PAF: 65%  Heart failure: NR  Prior stroke/TIA: NR  CAD: NR  Diabetes: NR  BMI: Recurrence: 31 (27-34); no recurrence: 28 (26-33) | First RF ablation. PVI. Additional ablation lines depending on underlying LVA and inducible LAMRT. | After ablation class I and III AADs not routinely initiated. Only patients with failed sinus rhythm restoration received previous AAD during blanking period and thereafter dependent on the rhythm in Holter ECG during follow-up. | Late arrhythmia recurrences were any atrial arrhythmia >30 s between 3 and 12 months after ablation. If electrical or pharmacologic cardioversion and/or repeat procedure needed after 3 months blanking period,  this was also considered as an arrhythmia recurrence, i.e. study endpoint. | 7-day Holter ECG recordings were performed at 3, 6 and 12 months. Additional ECGs and Holter ECG recordings were obtained when patients’ symptoms were suggestive of AF. | 12 months |
| Kornej 2018  COHORT 2  FT |  |  | Retrospective study. Heart Centre Leipzig, Germany, AF Ablation registry  (sub-sample of study cohort Kornej 2015)  n=873 | Age: Recurrence: 63 (55-70); no recurrence: 61 (54-68)  Male:64%  PAF: 65%  Heart failure: NR  Prior stroke/TIA: NR  CAD: NR  Diabetes: NR  BMI: Recurrence: 28 (26-31); no recurrence: 28 (25-31) | First RF ablation. PVI. Additional linear lesions at the LA roof, basal posterior wall and the LA (mitral) isthmus in patients with persistent AF. |  |  |  |  |

**Risk of bias assessment: population, predictors and outcomes (based on PROBAST)**

| **Study** | **Model** | | **Appropriate data source?** | **In/exclusions of participants appropriate?** | **Predictors defined and assessed in similar way for all participants?** | **Predictor assessment made without knowledge of outcome data?** | **All predictors available at the time the model is intended to be used?** | **Was the outcome determined appropriately?** | **Pre-defined/standard outcome definition?** | **Were predictors excluded from the outcome definition?** | **Was the outcome defined and determined in a similar way for all participants?** | **Was the outcome determined without knowledge of predictor information?** | **Appropriate time interval between predictor and outcome assessment?** |
| --- | --- | --- | --- | --- | --- | --- | --- | --- | --- | --- | --- | --- | --- |
| *Kosiuk 2015*  *DEV (LVA)* | **DR-FLASH** | **DEV √**  INT VAL X  **EXT VAL √**  U/M X  COMP X | NEI | Patients with previous catheter ablation or cardiac surgery excluded. Recurrence cohort did not include any patients who had PVI with substrate modification. | PY | NI | Y | PY | Y | Y | PY | NI | N/A |
| *Kosiuk 2015*  *VAL (LVA)* |  |  | NEI |  | NI | NI | Y | PY | YI | Y | PY | NI | N/A |
| Kosiuk 2015  VAL (recurrence) |  |  | PY |  | PY | PY | Y | PY | NI | Y | NI | NI | Y |
| Kornej 2018  COHORT 1 | **APPLE, MB-LATER, DR-FLASH** | DEV X  INT VAL X  **EXT VAL √**  U/M X  **COMP √** | PY | Exclusion criteria: pregnancy,  age <18 or >75, valvular AF, cancer, acute or systemic inflammatory diseases. Patients included on basis of available variable data. | PY | PY | Y* | Y | Y | Y | PY | NI | Y |
| Kornej 2018  COHORT 2 |  |  | PY |  | PY | PY | Y* | Y | Y | Y | PY | NI | Y |

* MB-LATER score includes early recurrence as a variable for prediction of late recurrence, so score cannot be used pre-procedurally

**Risk of bias assessment: analysis (based on PROBAST)**

| **Study** | **Model** | **Was there a reasonable number of participants with the outcome?** | **Were continuous and categorical predictors handled appropriately? For validation: was model evaluated as originally fitted?** | **Were all enrolled participants included in the analysis?** | **Were participants with missing data handled appropriately?** | **Was selection of predictors based on univariate analysis avoided? (DEV only)** | **Were complexities in the data (e.g. censoring, competing risks, sampling of control participants) accounted for appropriately?*** | **Were relevant model performance measures evaluated appropriately?** | **Were model overfitting and optimism in model performance accounted for? (DEV only)** | **Do predictors and their assigned weights in the final model correspond to the results from the reported multivariable analysis? (DEV only)** | **Where applicable: Appropriate quantification of added value (0ne score compared to another)? Appropriate method of updating model?** |
| --- | --- | --- | --- | --- | --- | --- | --- | --- | --- | --- | --- |
| Kosiuk 2015  DEV (LVA) | **DR-FLASH**  **DEV √**  INT VAL X  **EXT VAL √**  U/M X  COMP X | N  12 candidate variables, 66 events, <10 EPV | PN  Appears cut-offs based on study data | NI | NI | N  Based on group comparisons. | N/A | N  No calibration measures. | N  No details on internal validation. | N  1 point assigned for each statistically significant variable (based on group comparisons). | N/A |
| Kosiuk 2015  VAL (LVA) |  | N  44 events. | Y  Score applied as developed. | NI | NI |  | N/A  No model re-fitting. | N  No calibration measures. |  |  | N/A |
| Kosiuk 2015  VAL (recurrence) |  | Y  158 events | Y  Score applied as developed. | NI | NI |  | N/A  No model re-fitting. | N  No discrimination or calibration measures. |  |  | N/A |
| Kornej 2018  COHORT 1 | **APPLE, MB-LATER, DR-FLASH**  DEV X  INT VAL X  **EXT VAL √**  U/M X  **COMP √** | N  64 events | Y  Score applied as previously defined. | NI  NB Patients included on basis of available variable data. | NI |  | N/A  No model re-fitting | N  No calibration measures |  |  | No measures that quantify added benefit of using one score over another. |
| Kornej 2018  COHORT 2 |  | Y  300 events |  |  |  |  |  |  |  |  |  |

**Results**

| **Study** | **Model** | **% AF recurrence** | **Discrimination measure**  **C-statistic/**  **AUC** | **Calibration measure** | **Sensitivity/**  **specificity**  **(threshold)** | **Other measures reported** | **NRI/NDI** | **Other measures relating to model updating/**  **comparisons** |
| --- | --- | --- | --- | --- | --- | --- | --- | --- |
| Kosiuk 2015  (Cohort for predicting recurrence only) | **DR-FLASH**  **DEV √**  INT VAL X  **EXT VAL √**  U/M X  COMP X | 158/484 (33%) | NR | NR | NR | OR 1.311 (95% CI 1.13, 1.52) for every point increase on score.  OR 1.776 (95% CI .093, 2.883) for score >3. | N/A | N/A |
| Kornej 2018  COHORT 1 | **APPLE, MB-LATER, DR-FLASH**  DEV X  INT VAL X  **EXT VAL √**  U/M X  **COMP √** | 64/241 (27%) (NB states 62 in table) | NR | NR | NR | OR APPLE 1.063 (0.831-1.358, p= 0.627); adjusted OR APPLE 0.967 (0.706-1.326, p= 0.837)  OR MB-LATER 1.373 (1.040-1.811, p= 0.025); adjusted OR MB-LATER 1.445 (1.028-2.030, p= 0.034)  OR DR-FLASH 1.151 (0.944-1.403, p= 0.165); adjusted OR DR-FLASH 0.959 (0.751-1.226, p= 0.740) | NR | NR |
| Kornej 2018  COHORT 2 |  | 300/873 (34%) | APPLE 0.638 (0.599, 0.676), DR-FLASH 0.662 (0.624, 0.70)  MB-LATER 0.567 (0.526, 0.607) | NR | NR | OR APPLE 1.747 (1.527-1.998, p <0.001); adjusted OR APPLE 1.550 (1.333-1.803, p <0.001)  OR MB-LATER 1.550 (1.333-1.803, p <0.001); adjusted OR MB-LATER 1.747 (1.527-1.998, p <0.001)  OR DR-FLASH 1.310 (1.186-1.447, p <0.001); adjusted OR DR-FLASH 1.242 (1.118-1.381, p= 0.001) | NR | NR |

1. Kosiuk J, Dinov B, Kornej J, et al. Prospective, multicenter validation of a clinical risk score for left atrial arrhythmogenic substrate based on voltage analysis: DR-FLASH score. *Heart Rhythm* 2015;12(11):2207-12. doi: 10.1016/j.hrthm.2015.07.003 [published Online First: 2015/07/07]

2. Kornej J, Schumacher K, Dinov B, et al. Prediction of electro-anatomical substrate and arrhythmia recurrences using APPLE, DR-FLASH and MB-LATER scores in patients with atrial fibrillation undergoing catheter ablation. *Scientific Reports* 2018;8(1):12686. doi: <https://dx.doi.org/10.1038/s41598-018-31133-x>
