# Supplementary material for: Predicting recurrent atrial fibrillation after catheter ablation: a systematic review of prognostic models
Source: Europace. 2020 Mar 30;22(5):748–60. doi: 10.1093/europace/euaa041 (PMC7203634; doi:10.1093/europace/euaa041)
Supplement: euaa041_Supplementary_Data [file euaa041_supplementary_data.zip › euaa041-suppl_data/Suppl file 5 Main study characteristics.docx]

**Main study characteristics**

| **Study** | **Model** | | **Sample** | **Population characteristics** | **Ablation procedure** | **Antiarrhythmic drugs (AAD)** | **Outcome definition** | **Method of outcome assessment** | **Length of Follow-up** |
| --- | --- | --- | --- | --- | --- | --- | --- | --- | --- |
| Berkowitsch 2012 | **“Risk score”**  **DEV √**  INT VAL X  EXT VAL X  U/M X  COMP X | Development of “risk score”; no validation. | Cohort of consecutive patients;  Kerckhoff Heart Centre, Germany;  n=702 | Age: 58 (50-65)  Male: 71%  PAF: 59.23%  Cardiomyopathy: 3.28%  Prior stroke/TIA: NR  CAD: 8.83%  Diabetes: 6.55%  BMI: 26.96 (24.82, 29.94) | First ablation.  RF or CB ablation at discretion of physician. PVI. Step-wise approach to targeting other sites if PVI insufficient. | AAD at discretion of investigator post-procedure. Discontinuation after 6 weeks if no recurrent AT. Re-administration if AF recurrence within first 3 months. | AF/left atrial tachycardia recurrence >30s after 3 month period. | ECG and 7-day Holter recordings before discharge, at 3, 6, 9, 12 months and then 6-monthly intervals.  Additional ECG in case of arrhythmia-related symptoms. | Median (IQR) 15.6 months (12.7, 42.3) |
| Wojcik 2013 | **ALARMEc**  **DEV √**  INT VAL X  EXT VAL X  **U/M √**  COMP X | One variable added to “risk score” (Berkowitsch 2012) and one variable cut-off (NLA) changed; new/modified version named ALARMEc and applied to repeat ablation cohort. | Cohort of consecutive patients who had CA between 2005 and 2008; Kerckhoff Heart Centre, Germany  n=213  Possible overlap with above cohort. | Age: 56 (47-64)  Male: 73%  PAF: 46.48%  Cardiomyopathy: 5.16%  Prior stroke/TIA: NR  CAD: 7.04%  Diabetes: 3.76%  BMI: 26.83 (24.69, 29.70) | Repeat RF ablation. PVI. If AF continued, stepwise approach including mitral isthmus line, roof line, CFAE ablation and isolation of superior vena cava in same procedure. | AADs discontinued at least 3 days before ablation. | AF, atrial flutter or atrial tachycardia lasting >30s, documented on any kind of ECG. | Follow-up at 3, 6, 9 and 12 months, then yearly. 7-day Holter recordings and a 12-channel ECG during each follow-up visit. Additional ECG in case of arrhythmia-related symptoms. | Median 24 months; IQR 9.5–39 months; max 60, min 6 months |
| Wojcik 2014 | **ALARMEc**  DEV X  INT VAL X  **EXT VAL √**  **U/M √**  COMP X | Validation of ALARMEc score in ablation cohort; cut-off for NLA as in Berkowitsch 2012 so effectively different model again. | Retrospective analysis of consecutive patients who had CA between 2005 and 2008; Kerckhoff Heart Centre, Germany  n=911  Possible overlap with above cohort. | Age: 59 (50; 65)  Male: 69.15%  PAF: 57.63%  Cardiomyopathy: 3.29%  Prior stroke/TIA: NR  CAD: 8.12%  Diabetes only: 0.66% (diabetes and hypertension: 6.48%)  BMI: 26.88 (24.93, 29.98) | RF (61%) or CB (39%) ablation. 27% with repeat ablation. PVI. If AF continued, stepwise approach including mitral isthmus line, roof line, CFAE ablation and isolation of superior vena cava in same procedure. | AADs discontinued at least 3 days before ablation. | End point for analysis was maintenance  of sinus rhythm at follow-up time of 60 months. | Follow-up at 3, 6, 9 and 12 months, then yearly. Seven-day Holter--ECG at each follow-up visit. ECG in case of any palpitations. | 5 years |
| Wojcik 2015 | **ALARMEc**  DEV X  INT VAL X  **EXT VAL √**  U/M X  COMP X | Validation of ALARMEc score in CB ablation cohort. Score/cut-off as in Wojcik 2014. | Cohort of consecutive patients who had CA between 2005 and 2008; Kerckhoff Heart Centre, Germany  n=378  Possible overlap with above cohort. | Age: 58 (49; 65)  Male: 64.55%  PAF: 84.65%  Cardiomyopathy: NR  Prior stroke/TIA: NR  CAD: 6.88%  Diabetes: 5.03%  BMI: 26.71 (24.51, 29.55) | First CB ablation. PVI. | AADs discontinued at least 3 days before ablation. | Atrial fibrillation, atrial flutter or atrial tachycardia  recurrence after 3 months in the absence of AADs. | Follow-up at 3, 6, 9 and 12 months, then yearly. Seven-day Holter--ECG at each follow-up visit. ECG in case of any palpitations. | 12 months |
| Kornej 2015 | **APPLE**  DEV X  INT VAL X  **EXT VAL √**  U/M X  COMP X | Development of APPLE score based on variables identified in previous study (Kornej 2014) but no development study identified. Application of score in two cohorts. | Retrospective study;  Heart Centre Leipzig AF Ablation registry, Germany  n=1145  Sub-set of cohort from Kornej 2014. | Age: 60 (10)  Male: 65%  PAF:62 %  Heart failure: 7%  Prior stroke/TIA: 8%  CAD: 15%  Diabetes: 17%  BMI: 29 (5) | First ablation. RF ablation in 34.27%, CB in 65.73%. PVI. Additional linear lesions at LA roof, basal posterior wall and LA (mitral) isthmus in patients with persistent AF. | All class I or III AADs (except amiodarone) discontinued at least 5 half-lives before procedure. | Any atrial arrhythmia lasting >30s between 3 and 12 months after ablation. Also considered as recurrence: if electrical or pharmacological cardioversion and/or repeat procedure needed after 3 months. | Holter ECG at 3, 6 and 12 months. Additional ECG on symptom occurrence. | At least 12 months. |
|  |  |  | Retrospective study;  Vanderbilt AF Ablation Registry (US)  N=261 | Age: 61 (10)  Male:71%  PAF: 48 %  Heart failure: 10%  Prior stroke/TIA: 6%  CAD: 18%  Diabetes: 18%  BMI: 32 (7) | First ablation. PVI. Linear ablation in patients with persistent AF. CFAE ablation in patients with persistent AF according to operator discretion. | AADs continued peri-procedurally at discretion of the individual operator and discontinued 3 months after the procedure. |  |  |  |
| Furukawa 2016  ***Conf abstract*** | **“Risk score”, APPLE**  **DEV √**  INT VAL X  **EXT VAL √**  U/M X  COMP X | Development of new risk score and application of APPLE score in an ablation cohort.  *NB risk score not considered as not validated* | Consecutive patients; Osaka General Medical Centre, Japan.  n=511 | Age: 68.5 (10.2)  Male: NR  PAF: NR  Heart failure: NR  Prior stroke/TIA: NR  CAD: NR  Diabetes: NR  BMI: NR | First RF ablation. 16% with repeat ablation. | NR | Any atrial arrhythmia lasting more than 30s. | NR | Mean 438 (289 days) |
| Kornej 2017 | **APPLE**  DEV X  INT VAL X  **EXT VAL √**  U/M X  COMP X | Validation of APPLE score in a repeat CA cohort. | Retrospective study;  Heart Centre Leipzig AF Ablation registry, Germany  (sub-sample of study cohort Kornej 2015)  n=379 | Age: 60 (10)  Male:66%  PAF: 65%  Heart failure: NR  Prior stroke/TIA: NR  CAD: NR  Diabetes: 13%  BMI: 29 (4.8) | Repeat RF ablation (≥2). Assessment of previous PVI and linear ablation and gaps closed where necessary. | Class I and III antiarrhythmic medication not reinitiated after ablation. | Any atrial arrhythmia lasting >30s and occurring between 3 and 2 months after ablation. | Holter ECG at 3, 6 and 12 months. Additional ECG on symptom occurrence. | At least 12 months. |
| Kornej 2018  COHORT 1 | **APPLE, MB-LATER, DR-FLASH**  DEV X  INT VAL X  **EXT VAL √**  U/M X  **COMP √** | Validation of APPLE, MB-LATER and DR-FLASH scores in 2 cohorts.  *NB DR FLASH score not considered as not validated.* | Retrospective study.  BioAF cohort (Heart Centre Leipzig, Germany)  n=241  Possible overlap with above cohort. | Age: Recurrence: 64 (56-69); no recurrence: 65 (58-73)  Male:67%  PAF: 65%  Heart failure: NR  Prior stroke/TIA: NR  CAD: NR  Diabetes: NR  BMI: Recurrence: 31 (27-34); no recurrence: 28 (26-33) | First RF ablation. PVI. Additional ablation lines depending on underlying low voltage area and inducible left atrial macroreentry tachycardia. | After ablation class I and III AADs not routinely initiated. Only patients with failed sinus rhythm restoration received previous AAD during blanking period and thereafter dependent on the rhythm in Holter ECG during follow-up. | Late arrhythmia recurrences were any atrial arrhythmia >30 s between 3 and 12 months after ablation. If electrical or pharmacologic cardioversion and/or repeat procedure needed after 3 months blanking period,  this was also considered as an arrhythmia recurrence, i.e. study endpoint. | 7-day Holter ECG recordings were performed at 3, 6 and 12 months. Additional ECGs and Holter ECG recordings were obtained when patients’ symptoms were suggestive of AF. | 12 months |
| Kornej 2018  COHORT 2 |  |  | Retrospective study. Heart Centre Leipzig, Germany, AF Ablation registry  (sub-sample of study cohort Kornej 2015)  n=873 | Age: Recurrence: 63 (55-70); no recurrence: 61 (54-68)  Male:64%  PAF: 65%  Heart failure: NR  Prior stroke/TIA: NR  CAD: NR  Diabetes: NR  BMI: Recurrence: 28 (26-31); no recurrence: 28 (25-31) | First RF ablation. PVI. Additional linear lesions at the LA roof, basal posterior wall and the LA (mitral) isthmus in patients with persistent AF. |  |  |  |  |
| Miake 2018 | **APPLE**  DEV X  INT VAL X  **EXT VAL √**  U/M X  COMP X | Validation of APPLE score in an ablation cohort. | Prospective cohort, consecutive patients at Tottori University Hospital, Japan  n=254 | Age: 65.5 (9.3)  Male:72.4%  PAF: 68.9%  Heart failure: 13.4%  Prior stroke/TIA: 9.1%  CAD: 5.1%  Diabetes: 12.6%  BMI: 23.6 (3) | First RF ablation. PVI. | AADs discontinued for more than five times their half-lives. | AF > 30 s off AADs. Not within 90 days of ablation procedure. | Physical examination, 12-lead ECG and 24-h Holter monitoring at 1, 2, 3, 6, 9, 12, 16, 20 and 24 months. Patients self-monitored their pulses daily and on any irregularity of pulse, event monitoring or 14-day Holter monitoring was performed. | 24 months |
| Jud 2019 | **APPLE, SUCCESS**  **DEV √**  INT VAL X  **EXT VAL √**  **U/M √**  **COMP √** | New score developed by adding one variable to the APPLE score; also external validation of APPLE score. | Analysis of patients undergoing CA between 2009 and 2014; University Heart Centre Zurich.  n=192 | Age: 61.8 (9.19)  Male:76.04%  PAF: 60.42%  Heart failure: 5.21%  Prior stroke/TIA: 10.42%  CAD: 10.42%  Diabetes: 8.33%  BMI: 27.7 (4.23) | Single (67%) or repeat (33%) ablation. RF ablation in 96%, cryoenergy or laser in 4%. PVI. Additional linear lesions or substrate modifications  performed at discretion of operator in patients  with persistent AF. | Antiarrhythmic drugs used after the  intervention if required at the discretion of the treating physician. | “Success” defined as lack of AF lasting >30 s in Holter ECGs and absence of arrhythmia symptoms. “Partial success” defined  as reduction of AF duration >90% in patients without clinically symptomatic AF. AF recurrence defined as “failure”. | Symptom assessment and ECG monitoring at 3, 6, 12, and 24  Months; Holter ECGs available In 83% of the patients. | Mean 19 (12) months; range 3–55 months; 74%  assessed at least 12 months and 36%  at least 24 months after the procedure. |
| Kornej 2019 | **APPLE, MB-LATER**  DEV X  INT VAL X  **EXT VAL √**  U/M X  **COMP √** | Validation of APPLE and MB-LATER scores in an ablation cohort | Retrospective analysis of consecutive patients undergoing CA between 2007 and 2011. Heart Centre Leipzig AF Ablation registry.  n=879  Possible overlap with above cohort. | Age: 61 (54-68)  Male: 64%  PAF: 61.43%  Heart failure: 7%  Prior stroke/TIA: NR%  CAD: 15%  Diabetes: 18%  BMI: 28 (25-31) | First RF ablation. PVI. In patients with  persistent AF, additional linear lesions were added at the LA roof, the basal posterior wall and the LA (mitral)  isthmus. | All class I or III antiarrhythmic medications with the exception  of amiodarone were discontinued at least 5 half-lives before the procedure. | Arrhythmia recurrences defined as early (within 3 months after  ablation) and late (LRAF, over 3 months period). Electrical or pharmacologic cardioversion and/or repeat procedure  required were also considered as arrhythmia recurrence. | 7-day Holter ECG recordings immediately, and at 3, 6 and 12 months after ablation, then every 6 months. Additional resting ECGs and Holter ECG recordings obtained when patients’ symptoms  were suggestive of AF. | Median follow-up 37 months with 95%  CI (35;39) |
| Mesquita 2018  DEV | **ATLAS**  **DEV √**  **INT VAL √**  EXT VAL X  U/M X  COMP X | Development of ATLAS score in random 50% sample of cohort and validation in other 50%. | Retrospective study of consecutive registry patients; two hospitals in Lisbon, Portugal  n=960 | Age: 58 (12)  Male: 69%  PAF: 76%  Heart failure: NR  Prior stroke/TIA: 2%  CAD: 5%  Diabetes: 7%  BMI: 26 (5) | First RF ablation. PVI. Additional CTI ablation if atrial flutter observed or previously documented. | AADs maintained in all patients for the first 3months after the procedure and then withdrawn if  there was no AF recurrence. | Documented AF was defined by the presence of at least one episode of AF lasting more than 30 s in any ECG, 24 h Holter monitoring or event-loop recording. | 12-lead ECG  and 24 h Holter monitoring on the 1st, 3rd, 6th, and 12th months postablation,  followed by yearly assessments. Patients were encouraged to  contact the department if they experienced symptoms of AF recurrence. | Mean 4.2 (2.7) years. |
| Mesquita 2018  VAL |  |  | n=974 | Age: 59 (11)  Male: 68%  PAF: 77%  Heart failure: NR  Prior stroke/TIA: 3%  CAD: 4%  Diabetes: 8%  BMI: 27 (5) |  |  |  |  |  |
| Canpolat 2013 | **BASE-AF**  **DEV √**  INT VAL X  EXT VAL X  U/M X  COMP X | Development of BASE-AF score. | Prospective cohort, consecutive patients. Turkish Cryoablation Registry.  n=236 | Age: 54.6 (10.45)  Male: 54%  PAF: 79.6%  Heart failure: NR  Prior stroke/TIA: NR  CAD: 9.7%  Diabetes: 13.5%  BMI: 25.8 (6.2) | First CB ablation. PVI. | AADs discontinued five half-lives before procedure. | AF of >30 s duration when assessed with ECG > 3 months following AF ablation. | ECG and 24 hour Holter monitoring at 1, 3, 6, 9 and 12 months, then every 6 months. Patients received an AF event recorder if reported symptoms suggestive of AF. | Median 20 months (12-30). |
| Winkle 2016 | **CAAP-AF**  **DEV √**  INT VAL X  **EXT VAL √**  U/M X  COMP X | Development of CAAP-AF score and subsequent application of score in another cohort. | Retrospective study of consecutive patients 2003-10. Sequoia Hospital, California.  n=1125 | Age: 62.3 (10.3)  Male: 71.2%  PAF: 30.9%  Heart failure: NR  Prior stroke/TIA: 6.9%  CAD: 13.0%  Diabetes: 8.9%  BMI: 29.5 (5.4) | First ablation, repeat ablation in small proportion (mean number of procedures between 1.23 (0.44) and 1.37 (0.55)).  PVI and linear ablation of the LA roof.  Additional ablation as required of: CTI and/or mitral isthmus, low posterior LA lines and LA CFAE, coronary sinus CFAE ablation, and/or superior vena cava isolation. | AADs discontinued at least five half-lives and amiodarone at least 3 months before ablation. Some patients treated with antiarrhythmic drugs and/or cardioverted in first 3 months after ablation. | AF, flutter or tachycardia >30s off antiarrhythmic drugs after a 3-month blanking period. | Daily transtelephonic ECG strips for 1–3 months after ablation, continuous monitoring at 3 and 12 months (24 - to 48-hour ECG recording before 2006, 7-to14-day ECG recording thereafter.)  Patients seen or contacted frequently from 3 to 12 months, then at least annually and arrhythmia records obtained from hospitals and referring physicians. ECG recorders reissued for any arrhythmia symptoms. Pacemaker data used when available. Patients taught to check pulse daily and to use pulse oximeters, fitness monitors, or smartphone apps when unable to feel their pulse. | Mean 2.5 (1.7) years |
|  |  |  | Prospective cohort of consecutive patients 2010-12.  Sequoia Hospital, California.  n=937 | Age: 64.9 (9.3)  Male: 68.1%  PAF: 31.6%  Heart failure: NR  Prior stroke/TIA: 8.0%  CAD: 16.0%  Diabetes: 14.4%  BMI: 29.1 (5.7) |  |  |  |  | Mean 1.8 (0.9) years |
| Sanhoury 2017 | **CAAP-AF**  DEV X  INT VAL X  **EXT VAL √**  U/M X  COMP X | Validation of the CAAP-AF score in a CB ablation cohort. | Retrospective study at one centre (Cardiac Arrhythmia Research Centre, Milan)  n=283 | Age: 59.8 (11.4)  Male: 80%  PAF: 92%  Heart failure: 3%  Prior stroke/TIA: NR CAD: 4%  Diabetes: 5%  BMI: 26.2 (SD NR) | First CB ablation. PVI. | AADs discontinued prior to ablation and re-administered throughout blanking period (first 3 months); afterwards, the therapy could be stopped/continued according to follow-up and preference of the referring electrophysiologist. | Any atrial arrhythmia that lasted at least 30 s after the end of the blanking period. | 48-Holter monitoring at 1, 3, and 6 months and every 6 months thereafter. Some patients received implantable loop recorder. Patients asked to record their 12-lead ECG whenever they experienced symptoms suggestive of AF. | Mean 18 (6) months |
| de Vos 2010 | **HATCH**  **DEV √**  INT VAL X  EXT VAL X  U/M X  COMP X | Development of HATCH score | Retrospective study. Sub-sample from consecutively enrolled patients at several centres in the Euro Heart survey registry; patients with known rhythm status at 1 year.  n=1219 | Age: 64 (13)  Male: 57%  First detected or PAF: 100%  Heart failure: 21%  Prior stroke/TIA:9% CAD: 32%  Diabetes: 15%  BMI: 27 (4) | Aim is not to predict recurrence post-ablation, but to predict progression from paroxysmal to persistent AF. Not a post-ablation population, but score subsequently used in post-ablation populations. | 52% on AAD at baseline | PAF at baseline becoming persistent or permanent AF at 1-year follow-up or first detected AF at baseline with spontaneous conversion to sinus rhythm during admission becoming persistent or permanent AF at 1-year follow-up. | No details. | 12 months. |
| Tang 2010  ***Conference abstract*** | **HATCH**  DEV X  INT VAL X  **EXT VAL *√***  U/M X  COMP X | Validation of the HATCH score in a post-ablation cohort. | Retrospective study of consecutive patients. Beijing An Zhen Hospital.  n=608 | Age: NR  Male: NR  PAF: NR  Heart failure: NR  Prior stroke/TIA:NR  CAD: NR  Diabetes: NR  BMI: NR | First ablation. PVI. | NR | NR | NR | Mean 474 (330) days |
| Tang 2012 | **HATCH**  DEV X  INT VAL X  **EXT VAL *√***  U/M X  COMP X | Validation of the HATCH score in a post-ablation cohort. | Retrospective study of consecutive patients. Beijing An Zhen Hospital.  n=488  Possible overlap with above cohort. | Age: 57 (12), 57 (11)  Male: 71.6%, 69.2%  PAF: 100%  Heart failure: 2.8%, 2.6%  Prior stroke/TIA:5.1%, 4.2% (NB embolic events)  CAD: NR  Diabetes: 12.5%, 13.5%  BMI: 25.7 (3.4), 25.0 (3.4)  With and without recurrence respectively. | First RF ablation. PVI. CTI ablation if atrial flutter documented before procedure. Repeat procedures in 19.9%. | All AADs except amiodarone discontinued for at least 5 half-lives. | Recurrence defined as occurrence of atrial tachyarrhythmia documented by Holter or ECG recording beyond 3 months after the ablation and off AADs. | 12-lead ECG and 24-hour Holter recording at 1, 3, 6 and 12 months and every 6 months thereafter, or if patient was symptomatic. Additional monthly telephone interviews. | Mean 823 (532) days; range 91-1790 days |
| Silva 2011  ***Conference abstract*** | **HATCH**  DEV X  INT VAL X  **EXT VAL *√***  U/M X  COMP X | Validation of the HATCH score in a post-ablation cohort. | Prospective cohort.  Electrophisiology, Universidade Federal de São Paulo  n=47 | Age: 57.2 (13.2)  Male: 66%  PAF: 100%  Heart failure: NR  Prior stroke/TIA:NR  CAD: NR  Diabetes: NR  BMI: NR | First ablation. PVI. | NR | Clinical and/or ECG recurrence. | Includes ECG. No further details. | Mean 35.2 (11.9) months |
| Schmidt 2014 | **HATCH**  DEV X  INT VAL X  **EXT VAL *√***  U/M X  COMP X | Validation of the HATCH score in a post-ablation cohort. | Prospective cohort of consecutive patients. University of Rostock.  n=449 | Age: 61.7 (10.1)  Male: 65.5%  PAF: 75.1%  Heart failure:17.8%  Prior stroke/TIA:8.9%  CAD: 23.6%  Diabetes: NR  BMI: NR | First RF ablation. PVI. Some repeat ablations, mean of 1.38 procedures. | Antiarrhythmic drugs were continued for 3 months and then discontinued if patients were free of any arrhythmia. | A recurrent arrhythmia was defined as any atrial arrhythmia longer than 30 s irrespective of symptoms. | Implanted event recorders or 4–7-day Holter ECGs were studied during follow-up visits at 3, 6, and 12 months and every 6 months thereafter. | Mean 12.7 (7.1) months after last procedure (some repeat ablations; mean 1.38 procedures) |
| Miao 2012  (part translated from Chinese) | **HATCH**  DEV X  INT VAL X  **EXT VAL *√***  U/M X  COMP X | Validation of the HATCH score in a post-ablation cohort. | Retrospective study of consecutive patients. First Affiliated Hospital of Dalian Medical University, China.  n=123 | Age: 60.7 (9.3); 57.5 (10)  Male: 57.5%; 61.4%  PAF: 45%; 67.5%  Heart failure: 17.5%; 3.6%  Prior stroke/TIA: 2.5%; 3.6%  CAD: 2.5%; 4.8%  Diabetes: 2.5%; 14.5%  BMI: 28.0 (3.5); 27.2 (3.4)  Recurrence/non-recurrence groups respectively. | First RF ablation. PVI. | Not translated. | Atrial tachyarrhythmia lasting more than 30 s after 3 months. | ECG or Holter monitoring | 12 months |
| Shaikh 2014  ***Conference abstract*** | **HATCH +OSA**  **DEV X/√**  INT VAL X  **EXT VAL *√***  **U/M *√***  COMP X | Validation of a modified HATCH score in a post-ablation cohort. | Retrospective study.  University of UMass Memorial Center (UMMC) AF Treatment Registry,.  n= 78 | Age: 60 (1.1)  Male: 61%  PAF: NR  Heart failure: NR  Prior stroke/TIA:NR  CAD: NR  Diabetes: NR  BMI: NR | First ablation. PVI. RF (82%), CB (18%). | NR | NR | Holter monitoring and in-office ECGs. | 6 months |
| Shaikh 2015 | **HATCH.**  **B-HATCH**  **DEV √**  INT VAL X  **EXT VAL *√***  **U/M *√***  **COMP √** | Validation of a modified HATCH score in a post-ablation cohort. | Retrospective cohort of consecutive patients.  UMass Memorial Center (UMMC) AF Treatment Registry,  n=161 | Age: 59 (30-78)  Male: 70%  PAF: 60%  Heart failure: 4.3%  Prior stroke/TIA:11.2%  CAD: 31%  Diabetes: 29.2%  BMI: 32.2 (6.2) recurrence, 32 (5.9) no recurrence | First ablation. PVI. CB or RF (most) determined by electrophysiologist. In patients with persistent AF, additional linear lesions added at the LA roof, the basal posterior wall, and the LA isthmus at the discretion of the performing electrophysiologist. | AADs generally discontinued at a routine 3-month follow-up visit if no symptomatic AF recurrences reported or seen on routine post- procedure ECGs. | AF on a 12-lead ECG or any AF lasting ≥ 20 seconds on a cardiac event monitor 3 or more months after ablation. | Multiple sources, including clinical notes, telemetry data, and 12-lead ECGs from the participant's index hospitalization and outpatient follow up visits at 1, 3 and 6 months. Additional ECGs and Holter recordings obtained if symptoms were suggestive of AF. If cardioversion or repeat procedure for AF were needed, this was also considered as an AF recurrence. | 6 months |
| Chen 2015 | **HATCH**  DEV X  INT VAL X  **EXT VAL *√***  U/M X  COMP X | Validation of HATCH score in a post-ablation cohort. NB Ablation for atrial flutter and no prior AF. | Retrospective cohort of consecutive patients. Henan Provincial People’s Hospital and Beijing Anzhen Hospital, China.  n=233 | Age: 65.8 (11.0) with AF, 61.7 (9.7) no AF  Male: 75% with AF, 79% no AF  PAF: N/A  Heart failure: 31% AF, 12% no AF  Prior stroke/TIA:12% with AF, 4% no AF  CAD: 24% with AF, m22% no AF%  Diabetes: 15%with AF, 18% no AF  BMI: 26.7 (3.5) with AF, 26.4 (3.5) no AF | CTI ablation for atrial flutter, repeat ablation in 5%. | All AADs except amiodarone discontinued for at least 5 half-lives. | New-onset AF during follow-up defined as symptomatic or asymptomatic AF documented by 12-lead ECG or 24 hour Holter monitoring that lasted at least 30 s. | 12-lead ECG and 24-hour Holter recordings performed at 3 and 6 months and every 6 months thereafter, or if patient was symptomatic. | Mean 29.1 (18.3) months |
| Garcia-Seara 2016 | **HATCH**  DEV X  INT VAL X  **EXT VAL *√***  U/M X  COMP X | Validation of HATCH score in CTI for atrial flutter cohort; for predicting AF. | Retrospective cohort including all patients admitted between 1998-2010. Hospital Clínico y Universitario de Santiago de Compostela, Spain.  n=408 | Age: mean 62-65.5  Male: 84%  Persistent atrial flutter: 50%; previous AF: 40%  Heart failure: 18.1%%  Prior stroke/TIA:7%  CAD: 17.1%  Diabetes: 20.8%  ‘Obesity’: 25.2% | RF ablation in 97.9% and irrigated-tip catheter 2.1%. CTI ablation for atrial flutter. 13 patients (3.2%) with AF during follow-up  who underwent PVI. | AAD therapy maintained after CTI ablation in patients with prior AF and withdrawn in patients with isolated atrial flutter. Longer maintenance of AAD therapy at discretion of the clinician. | The occurrence of AF was defined as documentation during ECG or ECG Holter monitoring of at least 30 s of AF. | FU visits every 6 months, and 24-h Holter monitoring every year. ECG each time a patient went to the emergency room or saw their GP. | Mean 5.9 ± 3.1 years (range 1.6–13.5 years). |
| Providencia 2017  ***Conference abstract*** | **HATCH, AF-FREEDOM**  **DEV *√***  INT VAL X  **EXT VAL *√***  U/M X  **COMP *√*** | External validation of HATCH score and comparison with a new score, AF-FREEDOM.  *NB AF-FREEDOM not considered as not validated.* | Single high-volume centre. No further details.  n=1293 | Age: NR  Male: NR  PAF: NR  Heart failure: NR  Prior stroke/TIA:NR  CAD: NR  Diabetes: NR  BMI: NR | First ablation. No further details. | NR | Freedom from atrial arrhythmia relapse after 3 months. | NR | Mean 12 months (IQR 6-12 months) |
| Mujovic 2017  DEV | **MB-LATER, APPLE, ALARMc, BASE-AF2, HATCH**  **DEV *√***  INT VAL X  **EXT VAL *√***  U/M X  **COMP *√*** | Development and internal validation of MB-LATER score, external validation of and comparison with APPLE, ALARMc, BASE-AF2, HATCH  Used for very late prediction of recurrence, i.e. after 12 months. | Retrospective analysis of consecutive patients. Clinical Centre of Serbia.  n=133 | Age: 56.9 (11.8)  Male: 63.9%  PAF: 69.2%  Heart failure: 13.5%  Prior stroke/TIA:7.5%  CAD: 5.3%  Diabetes: 9.8%  BMI: 27.5 (4.1) | First RF ablation (n=103) repeat RF ablation (n=30). PVI + additional stepwise ablation where required (linear ablation, CFAE ablation). | All AADs were discontinued for ≥ 5 half-lives before the procedure; amiodarone stopped > 1 month prior to ablation. | Post-procedural finding of any symptomatic or asymptomatic atrial tachyarrhythmia (AF, atrial tachycardia) and/or typical atrial flutter) lasting > 30 sec was considered the arrhythmia recurrence. | Physical examination, 12-lead ECG and 24-hour Holter-recording at discharge, 1, 3 and 6 months, and every 6 months thereafter. Patients instructed to obtain ECG in case of symptoms suggestive of arrhythmia recurrence. Additional non-invasive work-up in symptomatic patients, primarily by 24–48-hour Holter-monitoring and more extensive rhythm monitoring if needed, including 7-day Holter-monitoring, event recorder use for 2–4 weeks, exercise stress testing and hospital admission for a 2–3-day observation. | Mean 29.1 (10.1) months (13-48 months) |
| Mujovic 2017  VAL |  |  | Patients not included in development cohort due to short follow-up  n=39 | Age: 56.9 (10.9)  Male: 68%  PAF: 51.3%  Heart failure: 10.3%  Prior stroke/TIA:2.6%  CAD: 5.1%  Diabetes: 12.8%  BMI: 27.9 (4.6) | First RF ablation (n=32) repeat RF ablation (n=7). PVI + additional stepwise ablation where required (linear ablation, CFAE ablation). |  |  |  | Mean 14.6 (1.9 months), 13-17 months |
| Potpara 2019 | **MB-LATER, CAAP-AF**  DEV X  INT VAL X  **EXT VAL √**  U/M X  **COMP √** | Validation of MB-LATER and CAAP-AF score in a post-ablation cohort. | Retrospective analysis of consecutive patients.  John Hopkins Hospital, Baltimore.  n=226 | Age: 58.5 (52-65)  Male: 72.1%  PAF: 62.8%  Heart failure: 9.7%  Prior stroke/TIA:8.4%  CAD: 15%  Diabetes: 10%  BMI: 29.3 (25.1, 35.7) | RF ablation (CB ablation in 1%). Single ablation in 88.1%, repeat ablation in 11.9%. PVI in all. In patients with non-paroxysmal AF, additional substrate modification performed, including LA linear ablation (ablation of the roof line and mitral isthmus line or posterior box isolation) or the LA defragmentation (elimination of all CFAEs). | After the 3-month blanking period, AADs were generally discontinued. | Any symptomatic or asymptomatic electrocardiographically documented atrial tachyarrhythmia (AF, atrial tachycardia and/or atrial flutter) lasting >30 s after the procedure was considered “the AF recurrence”. | Physical examination, 12-lead ECG and 24-hour Holter ECG at discharge, and at 1, 3, 6 months and every 6 months thereafter. Additional ECG or 24-hour Holter in patients who complained of symptoms suggestive of arrhythmia relapse. | Median 2 years (IQR 1.1-4.1 years) |
| Kaplan 2018  ***Conference abstract*** | **MB-LATER**  DEV X  INT VAL X  **EXT VAL √**  U/M X  COMP X | Validation of MB-LATER score in a CB ablation cohort.  Used for *very late prediction* of recurrence, i.e. after 12 months. | Single centre. No further details.  n=69 | Age: 61.9 (9.7) low score group, 61.3 (12.5) high score group  Male: 62% low score group, 87% high score group  PAF: 95% low score group, 39% high score group  Heart failure: NR  Prior stroke/TIA:NR  CAD: NR  Diabetes: NR  BMI: NR | CB ablation. No further details. | NR | NR | NR | Median 2.1 years |
| Deng 2018 | **MB-LATER, APPLE, HATCH, BASE-AF2, CAAP-AF**  DEV X  INT VAL X  **EXT VAL √**  U/M X  **COMP √** | Validation of MB-LATER score in a CB ablation cohort and comparison with other scores. | Retrospective analysis of consecutive data. Guangdong General Hospital, China.  n=1423 | Age: 57.18 (11.6)  Male: 68%  PAF: 77.2%  Heart failure: 5.1%  Prior stroke/TIA: 6%  CAD: 7.5%  Diabetes: 10.1%  BMI: 24.5 (3.3) | RF or CB ablation. Single procedure. PVI in all. CTI, superior vena cava isolation (SVCI), linear ablations of LA roof or mitral isthmus, and  CFAE performed where necessary. | Amiodarone was stopped more than 1 month and other AADs were discontinued ≥5 half-lives before the procedure. | Arrhythmia recurrence was defined as any symptomatic or asymptomatic atrial tachyarrhythmia (AF, atrial tachycardia[AT] and/or atrial flutter[AFL]) lasting >30 seconds. | Physical examination, 12-lead ECG and 24-hour Holter ECG performed at discharge, at 1, 3, 6 months and every 6 months thereafter. Additional ECG or 24-hour Holter performed in patients who complained of symptoms suggestive of arrhythmia relapse. | Mean 20.7 (8.8 months) |
| Bavishi 2019 | **MB-LATER, APPLE, ALARMEc, BASE-AF2**  DEV X  INT VAL X  **EXT VAL √**  U/M X  **COMP √** | External validation and comparison of a range of scores. | Retrospective analysis of prospectively maintained registry. Northwestern Memorial Hospital Chicago.  n=542 | Age: 61.3 (10.5)  Male: 67.2%  PAF: 51.7%  Heart failure: NR  Prior stroke/TIA: 6.5%  CAD: 15.9%  Diabetes: 11.1%  BMI: 29.7 (6.3) | CB ablation. Repeat procedure in 13.8%. PVI. | In general, antiarrhythmic medications were stopped after  the blanking period | AF recurrence was  defined as AF > 30 s on monitoring without a requirement for AAD. | At minimum, a 3-week AF monitor at 3  months postprocedure, and 24- to 48-h Holter monitors thereafter at  6-month intervals up to 2 years, downloads from implanted devices,  and readings from smartphone monitors where available.  Additional monitoring  in response to patient symptoms. | At least 12 months.  Median 21.5 months. |
| Jarman 2012  ***Conf abstract***  Cohort 1 DEV | **“Simple score”**  **DEV √**  INT VAL X  **EXT VAL √**  U/M X  COMP X | Development of score based on clinical variables and testing of the score in separate cohort.  Persistent AF patients. | Retrospective analysis.  n=130 | Age: NR  Male: NR  PAF: 0%  Heart failure: NR  Prior stroke/TIA:NR  CAD: NR  Diabetes: NR  BMI: NR | Catheter ablation. No further details. | NR | Successful outcome defined as absence of any sustained atrial tachyarrhythmia after a 3 month blanking period. | NR | Mean 12 (3) months |
| Jarman 2012  ***Conf abstract***  Cohort 2 VAL |  |  | Prospective cohort.  n=275 | Age: NR  Male: NR  PAF: 0%  Heart failure: NR  Prior stroke/TIA: NR  CAD: NR  Diabetes: NR  BMI: NR |  |  |  |  | NR |
| Egami 2017 ***Conf abstract***  Cohort 1 DEV | **FER2CI score**  **DEV √**  INT VAL X  **EXT VAL √**  U/M X  COMP X | Development of a new score to predict very late recurrence (>12 months after ablation) and testing of this score in a separate cohort. | Retrospective analysis of consecutive patients.  n=112 | Age: 65 (8)  Male: 64%  PAF: 67%  Heart failure: NR  Prior stroke/TIA: NR  CAD: NR  Diabetes: NR  BMI: NR | First catheter ablation. No further details. | NR | Recurrence of AF >12 months after ablation. No further details. | NR | Mean 45 (16) months |
| Egami 2017 ***Conf abstract***  Cohort 2 VAL |  |  | No details  n=unclear | Age: 65 (11)  Male: 70%  PAF: 78%  Heart failure: NR  Prior stroke/TIA:NR  CAD: NR  Diabetes: NR  BMI: NR |  |  |  |  | Mean 27 (7) months |

CA=catheter ablation; CAD=coronary artery disease; CB=cryoballoon; CFAE=complex fractioned atrial electrogram; COMP=study which compares two or more models; CTI=cavotricuspid isthmus; DEV=model development study; EXT VAL= external validation of model; INT VAL=internal validation of model; LA=left atrial; NR=not reported; PAF=paroxysmal atrial fibrillation; PVI=pulmonary vein isolation; RF=radiofrequency; TIA=transient ischaemic attack; U/M= model update or modification.
